# Shared genetics and causal relationships between major depressive disorder and COVID-19 related traits: a large-scale genome-wide cross-trait meta-analysis

**DOI:** 10.3389/fpsyt.2023.1144697

**Published:** 2023-06-23

**Authors:** Ziqi Li, Weijia Dang, Tianqi Hao, Hualin Zhang, Ziwei Yao, Wenchao Zhou, Liufei Deng, Hongmei Yu, Yalu Wen, Long Liu

**Affiliations:** ^1^Department of Health Statistics, School of Public Health, Shanxi Medical University, Taiyuan, Shanxi, China; ^2^Department of Statistics, University of Auckland, Auckland, New Zealand

**Keywords:** major depressive disorder, COVID-19, cross-trait meta-analysis, Mendelian randomization, functional annotation

## Abstract

**Introduction:**

The comorbidity between major depressive disorder (MDD) and coronavirus disease of 2019 (COVID-19) related traits have long been identified in clinical settings, but their shared genetic foundation and causal relationships are unknown. Here, we investigated the genetic mechanisms behind COVID-19 related traits and MDD using the cross-trait meta-analysis, and evaluated the underlying causal relationships between MDD and 3 different COVID-19 outcomes (severe COVID-19, hospitalized COVID-19, and COVID-19 infection).

**Methods:**

In this study, we conducted a comprehensive analysis using the most up-to-date and publicly available GWAS summary statistics to explore shared genetic etiology and the causality between MDD and COVID-19 outcomes. We first used genome-wide cross-trait meta-analysis to identify the pleiotropic genomic SNPs and the genes shared by MDD and COVID-19 outcomes, and then explore the potential bidirectional causal relationships between MDD and COVID-19 outcomes by implementing a bidirectional MR study design. We further conducted functional annotations analyses to obtain biological insight for shared genes from the results of cross-trait meta-analysis.

**Results:**

We have identified 71 SNPs located on 25 different genes are shared between MDD and COVID-19 outcomes. We have also found that genetic liability to MDD is a causal factor for COVID-19 outcomes. In particular, we found that MDD has causal effect on severe COVID-19 (OR = 1.832, 95% CI = 1.037–3.236) and hospitalized COVID-19 (OR = 1.412, 95% CI = 1.021–1.953). Functional analysis suggested that the shared genes are enriched in Cushing syndrome, neuroactive ligand-receptor interaction.

**Discussion:**

Our findings provide convincing evidence on shared genetic etiology and causal relationships between MDD and COVID-19 outcomes, which is crucial to prevention, and therapeutic treatment of MDD and COVID-19.

## 1. Introduction

Major depressive disorder (MDD), the most prevalent mental illness in the world, is defined by a persistently depressed mood and is linked to severe morbidity and a high risk of suicide (1). The comorbidity of depressive disorders and coronavirus disease of 2019 (COVID-19) related traits, has been widely reported (2). There was a public health crisis worldwide caused by COVID-19 pandemic that is created by the severe acute respiratory syndrome coronavirus 2 (SARS-CoV-2). Emerging evidence suggests people with MDD have higher risk of COVID-19 infection, hospitalization and mortality (3). COVID-19 survivors can suffer from pain, discomfort, minor mobility in their body and nervousness, and depression for 12 months (4). These evidences suggest there are possibilities of shared etiologies between MDD and COVID-19 outcomes, including severe COVID-19, hospitalized COVID-19 and COVID-19 infection. The potential comorbidity and the genetic relationships between MDD and the consequences of COVID-19 infection can not only worsen the quality of life, but also increase the associated healthcare costs. Therefore, identifying the underlying genetic causes that lead to the comorbidity between MDD and COVID-19 outcomes is of great importance.

Traditional observational studies usually investigate comorbidity by gathering information on disease history and lab tests (e.g., routine blood test), but they provide limited insights on the causes of the comorbidity (5). It could be the case that the one condition is the early sign of the other (e.g., mild cognitive impairment and Alzheimer’s disease) and it could also be the case that both conditions shared similar clinical characteristics that are used to define disease (e.g., psychological disorders) (6, 7).

The recently developed large-scale genome-wide cross-trait meta-analysis has shown their advantages in investigating the underlying genetic causes for comorbid conditions (5). Cross-trait meta-analysis is used to find the pleiotropic genomic single nucleotide polymorphisms (SNPs) and the genes shared by two comorbid conditions. For example, the cross-trait meta-analysis has found that there are shared genetic mechanisms between polycystic ovary syndrome and obesity (8), and it also provided the novel insights into the potential shared genetic architectures among five ocular diseases (9). Cross-trait meta-analysis utilizes summary statistics from multiple large scale genome wide association studies (GWAS) to infer shared disease etiology, and it has improved power in detecting genetic variants with small to moderate effects as compared to single-trait-based analysis (10). In addition, the recently developed cross-trait meta-analysis focuses not only on genetic variants with effect sizes on the same directions for both comorbid conditions, but also those that are either only related to one condition or related to both conditions with effect sizes on different directions [e.g., ASSET (11)].

While cross-trait meta-analysis facilitates the understanding of comorbidity, it cannot be used to infer the causal relationships between the comorbid conditions by itself. The Mendelian randomization (MR) is a widely used design in inferring causal relationships. MR constructs instrumental variables (IVs) using selected genetic variants that satisfy the three core assumptions, including (1) genetic variants are robustly associated with the exposure, (2) there is no confounders of the genetic variants and the outcome, and (3) genetic variants affect the outcome only through the exposure. With valid IVs, MR can test the hypotheses about whether an exposure is causally related to the outcome. To date, MR has greatly facilitated the causal inference. For example, bidirectional MR has shown that there are no causal relationships between deficiency of vitamin D and nonalcoholic fatty liver disease, although a high correlation between the two conditions have been observed in many studies (12). MR analysis has demonstrated that SARS-CoV-2 viral infection is a causal factor for the increased risk of hypothyroidism (13). The comorbidity between MDD and COVID-19 has been widely observed, and MR analysis can be a valid tool to explore their causal relationships.

In this study, we conducted a comprehensive analysis using the most up-to-date and publicly available GWAS summary statistics to explore shared genetic etiology and the causality between MDD and COVID-19 outcomes. We first used genome-wide cross-trait meta-analysis to identify the pleiotropic genomic SNPs and the genes shared by MDD and COVID-19 outcomes, and then explore the potential bidirectional causal relationships between MDD and COVID-19 outcomes by implementing a bidirectional MR study design. We further conducted functional annotations analyses to obtain biological insight for shared genes from the results of cross-trait meta-analysis.

## 2. Method

### 2.1. Summary statistics from GWAS for MDD and COVID-19 outcomes

GWAS summary statistics of MDD (65,075 cases and 232,552 controls) were obtained from publicly accessible web sites (GWAS Catalog Available online: https://www.ebi.ac.uk/gwas/publications/34278373) (14). Three datasets were obtained from the COVID-19 HGI GWAS round 6 (Release Date: 7 June 2021) (15), including severe COVID-19 (A2, 8,779 very severe respiratory-confirmed cases and 1,001,875 controls, excluding 23andMe), hospitalized COVID-19 (B2, 24,274 hospitalized COVID-19 cases and 2,061,529 controls, excluding 23andMe) and COVID-19 infections (C2, 112,612 COVID-19 cases and 2,474,079 controls, excluding 23andMe). These three datasets reflect different aspects of the COVID-19: susceptibility to disease shown in C2 and severity of the disease were contained in datasets A2 and B2. The details of GWAS data sets used in this study are summarized in Supplementary Table S1.

SNPs with minor allele frequency > 1% were utilized for meta-analysis using a fixed-effect model. Ambiguous SNPs (AT, TA, CG and GC) were excluded. We restricted all genetic data to European population to reduce potential bias from population stratification. Since all data are GWAS summary statistics that are accessible to the general public, no additional ethical review is required.

### 2.2. Cross-trait meta-analysis

To detect pleiotropic SNPs that contribute to the genetic correlations between MDD and COVID-19 outcomes, we conducted a cross-trait meta-analysis at individual SNP level using association analysis based on subsets (ASSET) (11): https://git.bioconductor.org/packages/ASSET. ASSET first searches subsets separately for studies with positive and negative associations, and then combines association signals from the two directions using a chi-square test-statistic. Therefore, it can discover SNPs with effects going in the opposite directions. We used the fixed-effect methods implemented in ASSET v2.4.0, which allows for an exhaustively exploration of all possible subsets of GWAS inputs. SNPs with *p*-values less than 5 × 10^−8^ were considered statistically significant. These SNPs include those that are associated with one of the condition, both conditions with effect sizes on the same direction and those with effect sizes on the opposite directions. Finally, functional mapping and annotation of genome-wide association studies (FUMA) were used to map SNPs to genes.

### 2.3. Bidirectional Mendelian randomization analysis

To identify the causal relationships between MDD and COVID-19 outcomes, we conducted bidirectional MR analyses for each pair of exposure and outcome. (https://mrcieu.github.io/TwoSampleMR/articles/introduction.html). We used the SNPs that are associated with MDD and/or COVID-19 related traits as IVs and carried out bidirectional MR analysis with the inverse variance weighted approach (16). One of the core assumptions of valid IVs for MR is that the genetic variants should robustly related to the exposure. Therefore, similar to existing work (17–19), suggestive genome-wide significance level (i.e., *p* < 5 × 10^−6^) was used to select SNPs, which not only facilitates the detection of SNPs with robust associations but also allows for the acquisition of sufficient SNPs for MR analyses. In addition, SNPs with LD > 0.001 were pruned and a clumping distance of 250Kb was used. It is generally accepted that environmental factors barely affect the genotypes of the selected SNPs, and thus it can be considered that there are no confounders for the selected SNPs. For the MR analyses, we used the traditional fixed-effect inverse-variance weighted (IVW) method to evaluate the causal effect of exposure on outcomes (13). IVW is the most efficient and statistically powerful MR method, but its validity heavily depends on whether its model assumptions (e.g., valid IVs) are satisfied. In MR analyses, the presence of pleiotropy suggests the violation of the assumption of valid IVs (i.e., genetic variants affect the outcome only through the exposure). Therefore, sensitivity analysis was used to assess the robustness of the causal effect estimates, including heterogeneity test (20), MR Egger intercept method testing for bias from pleiotropy (21), and the leave-one-out test (22).

### 2.4. Functional annotation analysis

To better understand the genetic mechanisms underlying shared genes between MDD and COVID-19 outcomes, we conducted FUMA (23) using the results of cross-trait meta-analysis (https://fuma.ctglab.nl/). FUMA is available as an integrative web-based platform and its core functions are the SNP2GENE (23) and GENE2FUNC (24). We used SNP2GENE with its default settings to first annotate the biological functions of SNPs and then map them to genes based on the position, eQTL and chromatin interaction information. For the functional annotations, we focused on the SNPs with ASSET value of *p* less than 5 × 10^−8^ as well as those that are at high linkage disequilibrium with them (i.e., *r*^2^ ≥ 0.6). We annotated the selected SNPs based on functional Categories, Combined Annotation Dependent Depletion (CADD) scores, RegulomeDB scores and chromatin states using FUMA (23). The CADD score reflects the deleteriousness of SNPs that are predicted by 63 functional annotations. A CADD score of 12.37 or above indicates the most deleterious variants (25). The RegulomeDB score shows the regulatory functionality of SNPs that are determined based on the overlap of existing functional data annotation in GTEx v7 dataset. ChromHMM helps annotate the non-coding genome. FUMA uses the ChromHMM to predicts 15 categories based on 5 chromatin marks for 127 epigenomes, and the accessibility of genomic regions can be shown by the chromatin state (26). We further utilized GENE2FUNC to annotate the mapped genes within the biological contexts. In particular, we used heatmap to visualize their tissue-specific gene expression levels (e.g., brain, liver, and artery) using information from Genotype-Tissue Expression (GTEx) v7 dataset provided by GENE2FUNC. We further used the hypergeometric tests implemented in GENE2FUNC to evaluate if these mapped genes are overrepresented in the pre-determined differentially expressed gene (DEG) sets in specific tissue types, where the pre-determined DEG sets are obtained by comparing the normalized expression level of each gene from one tissue with all other issues in GTEx v7 dataset.

## 3. Results

### 3.1. Cross-trait meta-analysis results between MDD and COVID-19 outcomes

**Figure 1:**
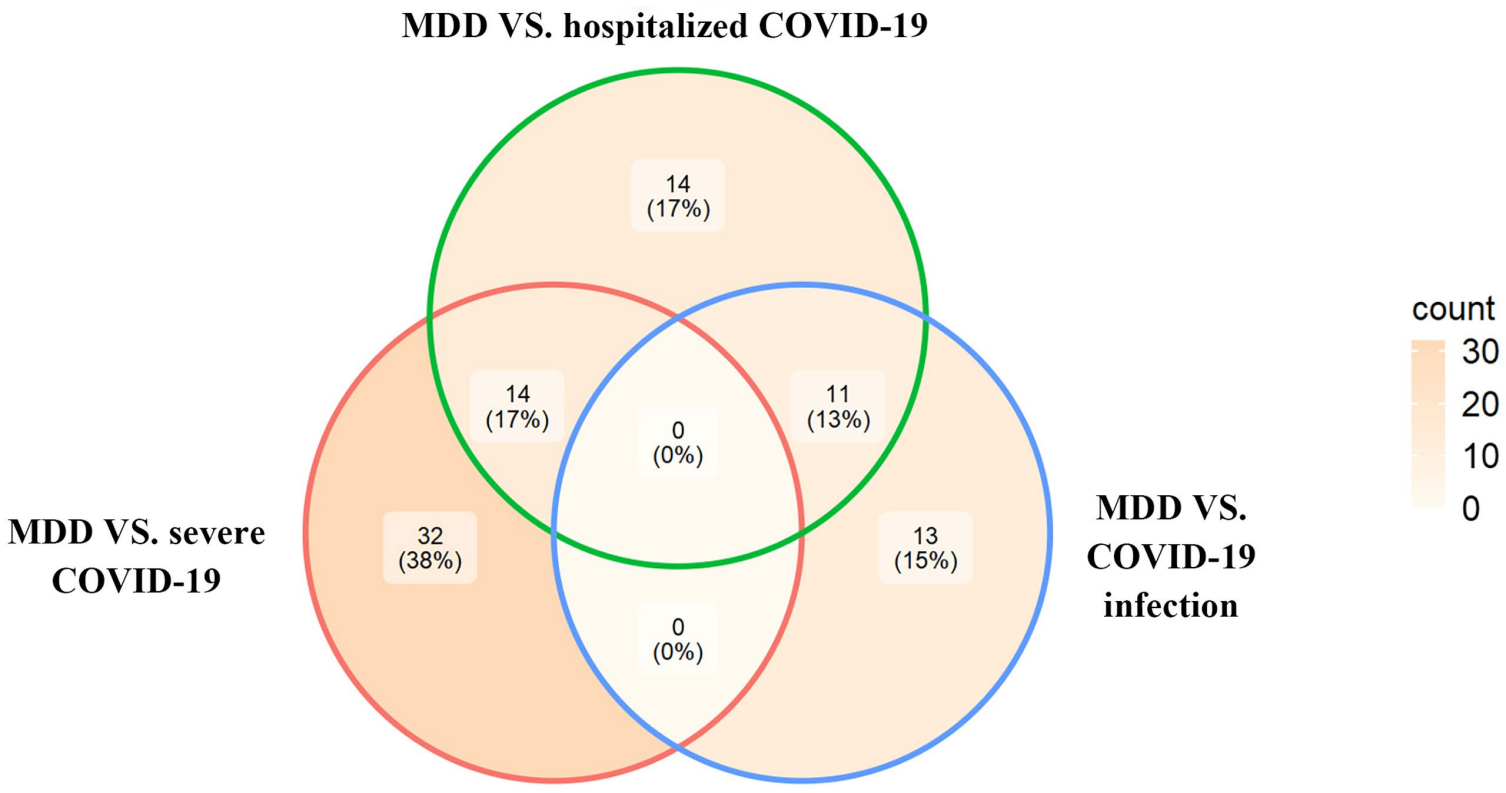
The Venn diagram of overlapping SNPs identified by the cross-trait meta-analysis across different trait pairs.

Based on the cross-trait meta-analysis, we identified 46 significant SNPs associated with MDD and severe COVID-19 located within 13 genes (Supplementary Table S2), 39 significant SNPs associated with MDD and hospitalized COVID-19 located within 11 genes (Supplementary Table S3) and 24 significant SNPs associated with MDD and COVID-19 infections located within 8 genes (Supplementary Table S4). We have found 14 overlapped significant SNPs between MDD/severe COVID-19 and MDD/hospitalized COVID-19, and 11 overlapped significant SNPs between the MDD/COVID-19 infection and MDD/hospitalized COVID-19 (Figure 1).

Notably, among the 46 significant SNPs that were shared between MDD and severe COVID-19 (Supplementary Table S2), the most significant one was rs2234358 (*P*_meta_ = 2.46 × 10^−11^) located within *FYCO1:CXCR6*, which is also shared by MDD and hospitalized COVID-19 (sentinel SNP [the most significant SNP]: rs17689471, *P*_meta_ = 1.48 × 10^−12^). Among the 39 SNPs that were shared between MDD and hospitalized COVID-19 (Supplementary Table S3), the most significant (rs17689471 at locus 17q21.31, *P*_meta_ = 1.48 × 10^−12^) and second significant SNPs (rs17689824 at locus 17q21.31, *P*_meta_ = 1.64 × 10^−12^) were both located at *CRHR1*. In addition, among the shared SNPs between MDD and COVID-19 infection (Supplementary Table S4), the most significant one (rs853676 at locus 6p22.1, *P*_meta_ = 2.74 × 10^−10^) was located at *ZSCAN31*.

### 3.2. Results from the bidirectional Mendelian randomization analysis

**Figure 2:**
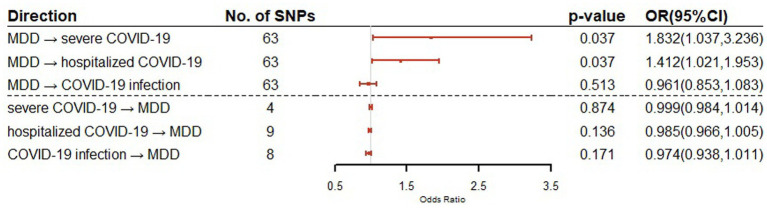
Bidirectional Mendelian randomization analysis of MDD and COVID-19 related traits using IVW method. Causal effect estimates are presented as odds ratios (OR) with 95% confidence intervals (CI).

For the effect of MDD on COVID-19 outcomes (severe COVID-19, hospitalized COVID-19 and COVID-19 infection), 63 SNPs were used as IVs in the bidirectional MR for each of the COVID-19 outcomes. For the effect of COVID-19 outcomes on MDD, a total of 4, 9, and 8 SNPs were used as IVs in the bidirectional MR for severe COVID-19, hospitalized COVID-19 and COVID-19 infection, respectively. The details of IVs were summarized in Supplementary Table S5. Our bidirectional MR analysis suggested that MDD has a genetical causal effect on COVID-19 outcomes. Notably, MDD confers a genetical causal effect on severe COVID-19 (IVW *β* = 0.6053, SE = 0.2902, *p* = 0.0370), and hospitalized COVID-19 (IVW *β* = 0.3451, SE = 0.1655, *p* = 0.0370), but not COVID-19 infection (IVW *β* = 0.0398, SE = 0.0608, *p* = 0.5125), as shown in Table 1. Conversely, no causal links were identified in the other direction from the MR analysis. The forest plot of the results of our bidirectional MR analysis is shown in Figure 2. The sensitivity analysis of our bidirectional MR analysis suggested that there is no significant heterogeneity (Supplementary Table S6) and pleiotropy (Supplementary Table S7), and the leave-one-out test also shows that the results are robust (Supplementary Figures S1–S6).

**Table 1:** Estimates of causal effect size between major depressive disorder and COVID-19 outcomes.

Phenotype 1	Phenotype 2	Direction	Causal effect size ± SE	*p*-value	No. of Instrumental variables
MDD	severe COVID-19	**→**	0.6053 ± 0.2902	0.0370	63
		**←**	−0.0012 ± 0.0075	0.8740	4
	hospitalized COVID-19	**→**	0.3451 ± 0.1655	0.0370	63
		**←**	0.0149 ± 0.01	0.1357	9
	COVID-19 infection	**→**	0.0398 ± 0.0608	0.5125	63
		**←**	−0.0265 ± 0.0193	0.1708	8

“→” refers to the phenotype 1 → phenotype 2 causal direction; “←” refers to the phenotype 2 → phenotype 1 causal direction.

### 3.3. Results from the functional annotation analysis

**Figure 3:**
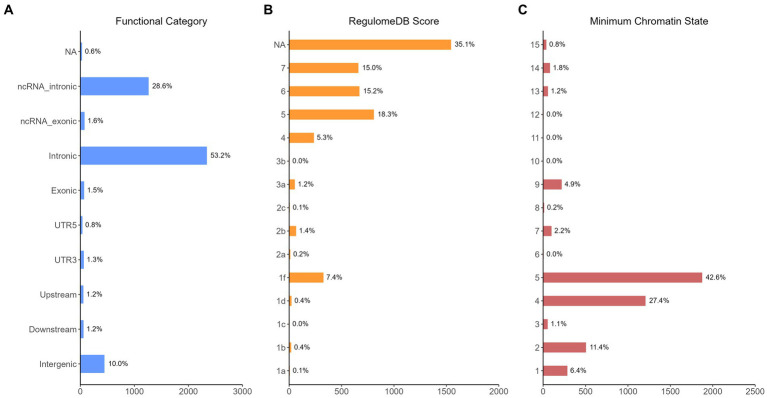
Distribution of the annotation for all SNPs jointly associated with the results of cross-trait meta-analysis of MDD and severe COVID-19. **(A)** Distribution of functional categories of SNPs in the shared genomic risk loci. **(B)** Distribution of RegulomeDB score for SNPs in shared genomic loci. **(C)** The minimum chromatin state across 127 tissue and cell types for SNPs in shared genomic loci, with lower states indicating higher accessibility and states 1–7 referring to open chromatin states.

**Figure 4:**
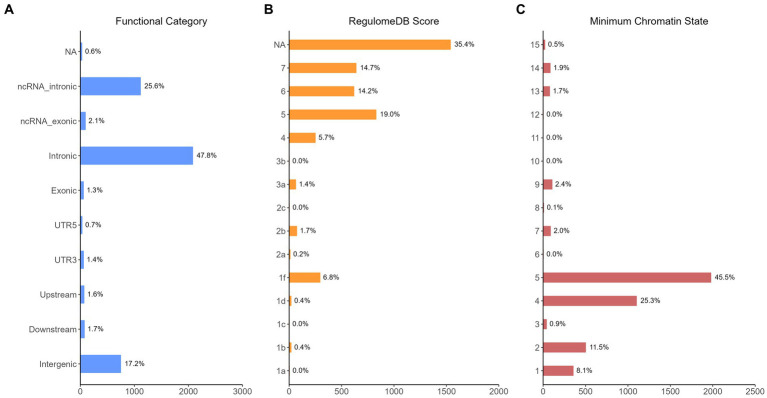
Distribution of the annotation for all SNPs jointly associated with the results of cross-trait meta-analysis of MDD and hospitalized COVID-19. **(A)** Distribution of functional categories of SNPs in the shared genomic risk loci. **(B)** Distribution of RegulomeDB score for SNPs in shared genomic loci. **(C)** The minimum chromatin state across 127 tissue and cell types for SNPs in shared genomic loci, with lower states indicating higher accessibility and states 1–7 referring to open chromatin states.

**Figure 5:**
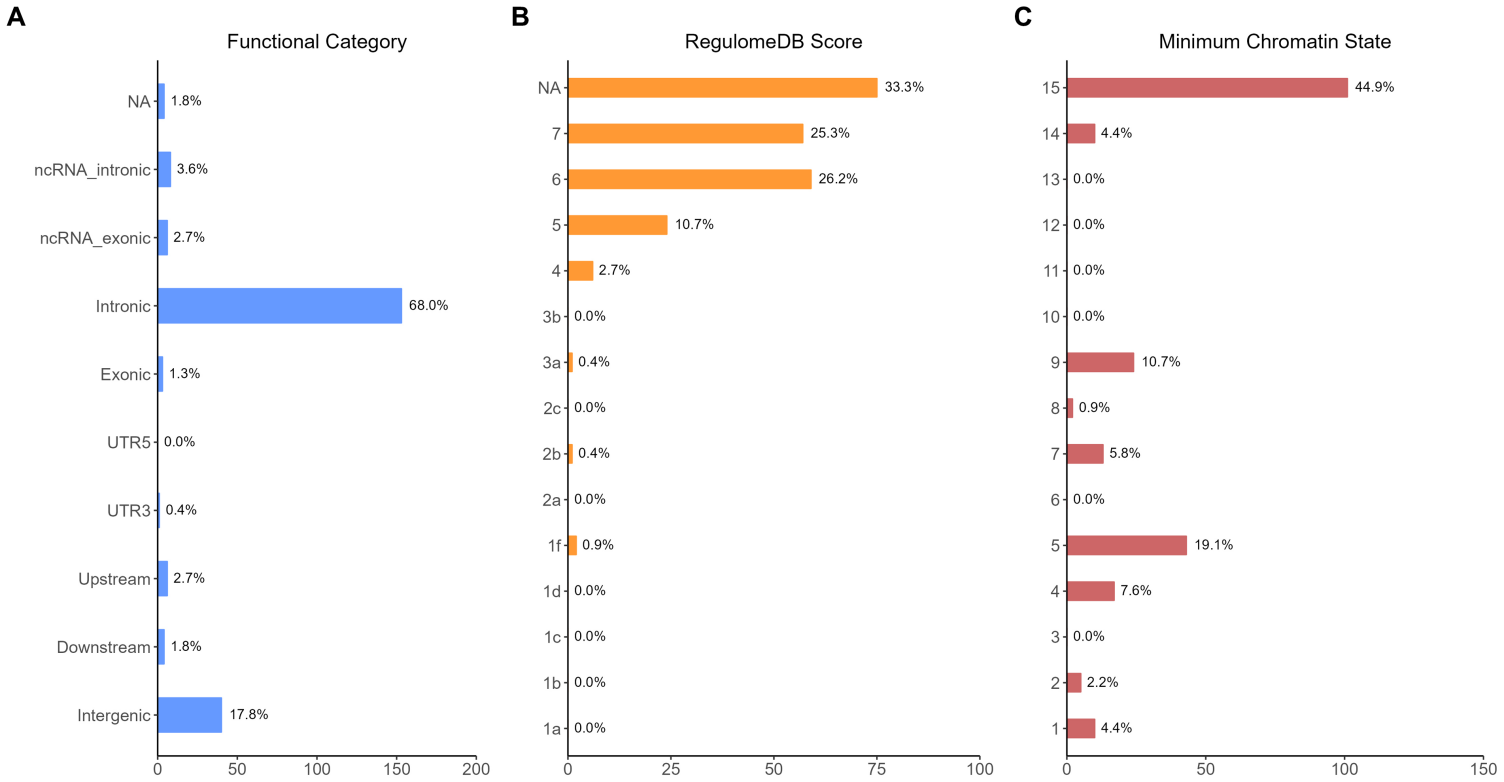
Distribution of the annotation for all SNPs jointly associated with the results of cross-trait meta-analysis of MDD and COVID-19 infection. **(A)** Distribution of functional categories of SNPs in the shared genomic risk loci. **(B)** Distribution of RegulomeDB score for SNPs in shared genomic loci. **(C)** The minimum chromatin state across 127 tissue and cell types for SNPs in shared genomic loci, with lower states indicating higher accessibility and states 1–7 referring to open chromatin states.

**Figure 6:**
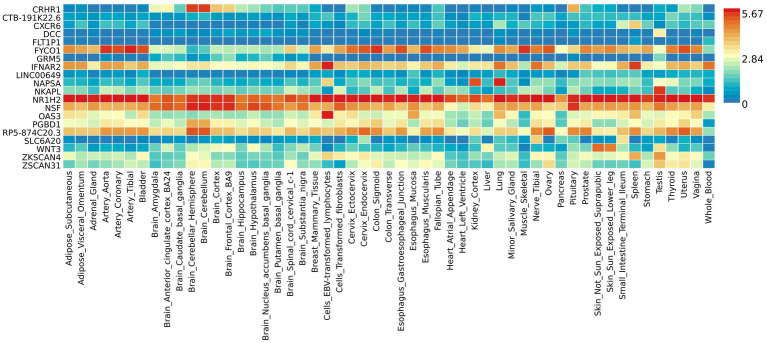
Shared genes expression heatmaps constructed with GTEx v7 (53 tissues). Genes and tissues are ordered by clusters for the GTEx heatmap. The abscissa represents the GTEx v7 tissues and the ordinate represents the genes selected by cross-trait meta-analyses.

**Figure 7:**
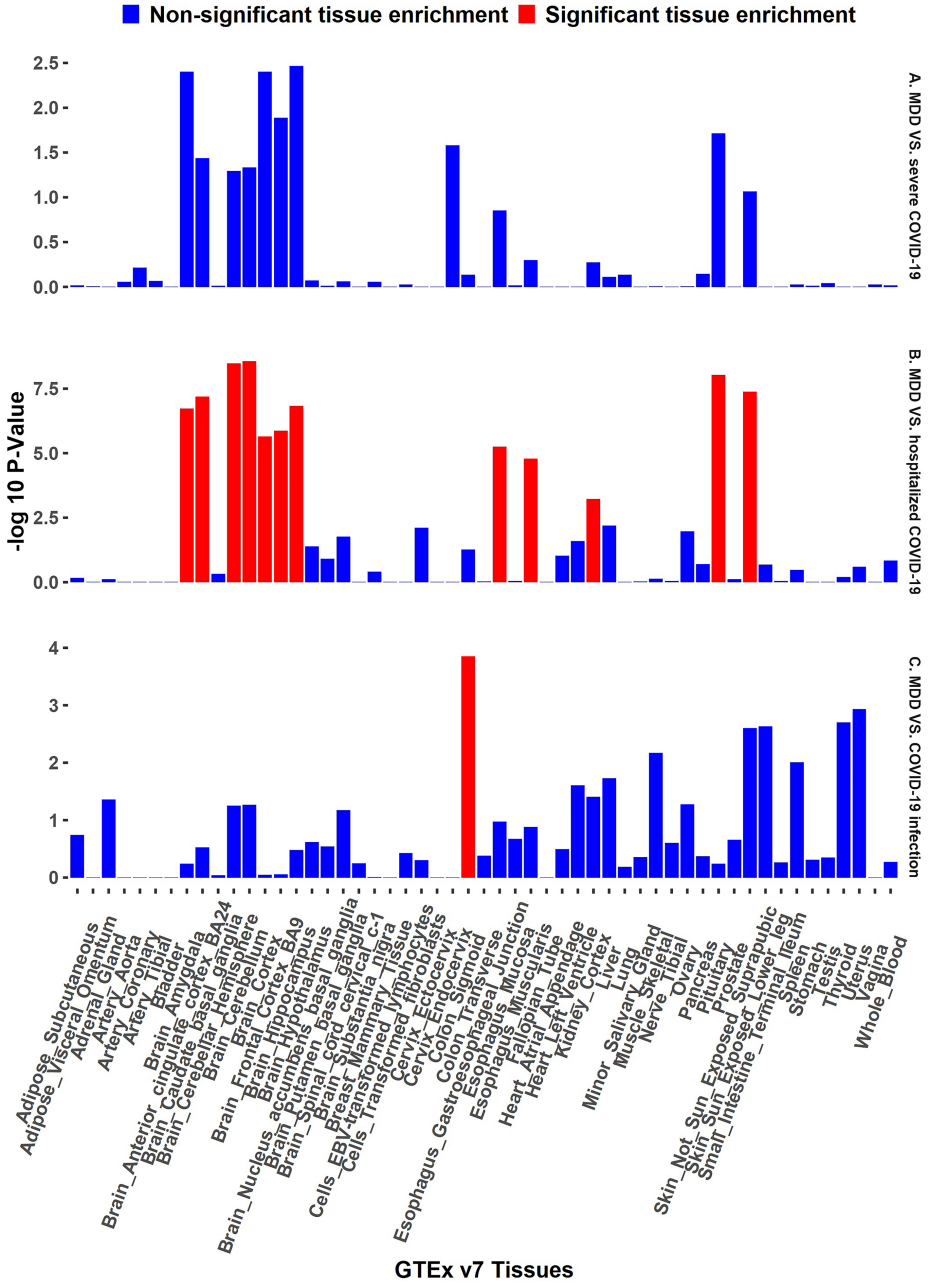
GTEx tissue enrichment analysis. Red bar represents significant tissue enrichment after Benjamin-Hochberg correction. The abscissa represents the GTEx v7 tissues and the ordinate represents the genes selected by cross-trait meta-analyses. **(A)** GTEx tissue enrichment analysis of MDD and severe COVID-19 (both side). **(B)** GTEx tissue enrichment analysis of MDD and hospitalized COVID-19 (both side). **(C)** GTEx tissue enrichment analysis of MDD and COVID-19 infection (both side).

The functional annotation analysis of all candidate SNPs selected based on the results of cross-trait meta-analysis between MDD and COVID-19 outcomes indicates that the SNPs are mostly intronic and intergenic. For SNPs selected from the cross-trait meta-analysis of MDD and severe COVID-19, 53.2, 10, and 1.5% of the candidate SNPs are intronic, intergenic and exonic, respectively (Figure 3A). For MDD and hospitalized COVID-19, 47.8, 17.2 and 1.3% of the candidate SNPs are intronic, intergenic and exonic (Figure 4A), respectively. Similarly, for MDD and COVID-19 infection, 68.0, 17.8 and 1.3% of the SNPs are intronic, intergenic and exonic (Figure 5A). For the most deleterious variants (i.e., CADD ≥ 12.37), we found one variant (rs199535), four SNPs (rs1679709, rs199439, rs199456 and rs199535) and 1 SNP (rs1679709) from the analysis of MDD and severe COVID-19, hospitalized COVID-19 and COVID-19 infections, respectively. The categories 1a-1f of RegulomeDB score indicate that variants are likely to affect binding and linked to the expression of a target gene. 8.3, 7.6, and 0.9% of the SNPs have RegulomeDB scores of 1a-1f for MDD and severe COVID-19, hospitalized COVID-19 and COVID-19 infection, respectively (Figure 3B, Figure 4B and Figure 5B). This indicates that SNPs in MDD and severe COVID-19, as well as hospitalized COVID-19, are more likely to have regulatory functions than those in MDD and COVID-19 infections. Notably, the most significant SNP (rs17689471) detected from the analysis of MDD and hospitalized COVID-19 has RegulomeDB score of 1 f. The distribution of minimum chromatin state showed that 91.1% of the candidate SNPs between MDD and severe COVID-19 (Figure 3C), 93.3% between MDD and hospitalized COVID-19 (Figure 4C) and 39.1% between MDD and COVID-19 infection (Figure 5C) are located in open chromatin states regions. The tissue-specific gene expression levels among genes, which are selected from eQTL mapping of the shared SNPs detected from our cross-trait meta-analysis, are shown in Figure 6. Notably, there are two genes (i.e., *NSF* and *NR1H2*) that exhibit higher expression levels among all tissue types. As shown in Figure 7, genes that are mapped through eQTL using significant SNPs shared by MDD and hospitalized COVID-19 are enriched in brain tissues, esophagus, kidney, pituitary and skin; genes that are mapped from SNPs associated with MDD and COVID-19 infection are enriched in colon; and genes that are obtained from SNPs that are detected from the analysis of MDD and severe COVID-19 are not enriched in any tissues.

## 4. Discussion

Investigations on the underlying genetic architecture that links MDD and COVID-19 outcomes and the causality that underlies them can advance our understanding of the comorbidity between MDD and COVID-19, which can aid in drug development, early prediction, and personalized treatment (27–29). In this study, we have identified genetic variants in 25 genes that are associated with MDD and/or COVID-19, and we also found that genetic predisposition to MDD is a causal factor to the severity of COVID-19 outcomes. Our findings suggest that MDD patients form a vulnerable group of severe COVID-19, and they should be encouraged to get the vaccine. In addition, screening for MDD could be included in the COVID-19 treatment regimen, as improved management may be achieved with add-on psychological or psychiatric interventions for subgroups with severer depression (30).

From the large scale cross-trait meta-analysis, we have found that 25 genes are directly implicated by the three cross-trait groups (MDD and severe COVID-19, MDD and hospitalized COVID-19, MDD and COVID-19 infection). These genes include *FYCO1*, *CXCR6*, *SLC6A20*, *CRHR1*, *RP11-105 N13.4*, *DCC*, *RP1-71H24.1*, *OAS3*, *NR1H2*, *NAPSA*, *IFNAR2*, *AP000295.9*, *CTB-191 K22.6*, *FLT1P1*, *NSF*, *ZSCAN31*, *RP5-874C20.6*, *RP5-874C20.3*, *ZKSCAN4*, *LINC00649*, *NKAPL*, *PGBD1*, *Wnt3*, *ACTR3P3*, *GRM5*. Most of them have already been demonstrated to be novel risk factors for COVID-19 and/or MDD. For example, it has been established that Corticotropin-releasing-hormone receptor 1 (*CRHR1*), a key modulator of the stress response, plays a significant role in the pathophysiology of MDD. Several studies have confirmed the link between depression and polymorphisms in the Corticotropin-releasing hormone 1 receptor gene (*CRHR1:RP11-105 N13.4*) (31, 32). The incidence of severe COVID-19 and respiratory diseases were both decreased by an intronic variation in *CRHR1* (30). *ZSCAN31* also plays a positive role in human embryonic development. It is relevant to both epilepsy and depression (33), and involved in the development of multiple embryonic organs, including brain (34). *ZSCAN31* is related to PDZ-binding motif (TAZ) expression in hepatocellular carcinoma cells, and the targeting of *ZSCAN31* and TAZ could express a novel therapeutic approach in hepatocellular carcinoma cells (35). The selective reduction of *Wnt3* expression in the ventral hippocampus following chronic restraint stress (CRS) suggested that *Wnt3* plays some roles in CRS-induced depression-like behaviors (36). In addition, both *NSF* and *Wnt3* are confirmed as putative causal genes for COVID-19 severity (37). Our research provides fresh insight into the genetic susceptibility of MDD and COVID-19 outcomes. In addition to those novel genes, we also found genes (e.g., *ACTR3P3*) that are not reported previously, and they are worth further investigation. Some of these common genes can have consequences for treatment regimens for people who have both disorders.

Our bidirectional Mendelian randomization study demonstrated the causal effects of MDD on COVID-19 related traits. We showed for the first time that genetically predicted MDD is associated with an increased risk of severe COVID-19 (OR = 1.832, 95% CI = 1.037–3.236) and hospitalization COVID-19 (OR = 1.412, 95% CI = 1.021–1.953). This is consistent with the findings of large cohort studies that show patients with MDD have a higher risk of COVID-19 hospitalization and mortality. Given the increased risk of severe COVID-19 and hospitalization COVID-19, our findings suggest that patients with MDD should be encouraged to get vaccines (3). There are no causal relationships between MDD and COVID-19 infection, although shared genetic genes were detected.

The results from the genetic and functional annotations indicate that the genes mapped from the SNPs detected from cross-trait analysis play varied roles in COVID-19 related traits. A low RegulomeDB score indicates a higher likelihood of having a regulatory function. Notably, the rs199456 has a RegulomeDB score of 1b and its CADD score is 15.73. It is shows that the rs199456 may affect transcription factor binding and be deleterious. Through the functional annotation analysis, we found that *NSF* and *NR1H2* have shown high expression levels in all GTEx dataset tissues, such as artery, brain, and colon. Besides, the shared genes between MDD and hospitalized COVID-19 were enriched in a variety of tissues, including various brain tissues, pituitary, and kidney, all of which are known to play significant roles in regulating hormone and enzyme function. However, the shared genes between MDD and COVID-19 infection were enriched only in colon tissue, and there is no significant independent tissue expression for MDD and severe COVID-19.

There are some limitations in this study. Firstly, the majority of the data included in the analysis came from individuals of European ancestry, and thus reduces the generalizability of our findings to other ethnic groups. Secondly, we only included SNPs that reach the genome-wide significance level (*p* < 5 × 10^−8^) for the cross-trait meta-analysis, which can lead to the overlook of some of genetic variants with small-to-moderate effect sizes. Finally, we mainly focused on genetic mechanisms underlying the comorbidity between MDD and COVID-19, and environment and their interactions with genetic variants are not considered.

## 5. Conclusion

In conclusion, we have detected shared genes between MDD and COVID-19 outcomes, and found that MDD is a risk factor of severe COVID-19 and hospitalized COVID-19. To the best of our knowledge, our study is the first study that has shed light on the understanding of the mechanisms leading to the comorbidity between MDD and COVID-19. It can open up new pathways for future functional validations, disease prevention, and therapeutic treatment.

## Data availability statement

According to the subheading for GWAS summary data, the GWAS summary statistics data that were examined were acquired from worldwide research consortia and public repositories. Through the links and references given in this study, the data are publicly accessible and available online. The original contributions presented in the study are included in the article/Supplementary material, further inquiries can be directed to the correspondingauthors.

## Ethics statement

Ethical review and approval was not required for the study on human participants in accordance with the local legislation and institutional requirements. Written informed consent for participation was not required for this study in accordance with the national legislation and the institutional requirements.

## Author contributions

LL and YW conceived and designed the study, directed, and followed the entire study. ZL performed quality control for GWAS summary data, visualized, and summarized the results of our study. ZL and WD performed data analysis and compared methods we studied. ZL wrote the first draft of the article and LL revised it. YW provided concrete practical advice and corrections on article writing. ZL, WD, TH, HZ, ZY, WZ, LD, HY, YW, and LL were involved in manuscript writing. All authors contributed to the article and approved the submitted version.

## Funding

This project is funded by the National Natural Science Foundation of China (Award nos. 81903418, 82173632, and 82273742), Early Career Research Excellence Award from the University of Auckland and the Marsden Fund from Royal Society of New Zealand (Project no. 19-UOA-209).

## Conflict of interest

The authors declare that the research was conducted in the absence of any commercial or financial relationships that could be construed as a potential conflict of interest.

## Publisher’s note

All claims expressed in this article are solely those of the authors and do not necessarily represent those of their affiliated organizations, or those of the publisher, the editors and the reviewers. Any product that may be evaluated in this article, or claim that may be made by its manufacturer, is not guaranteed or endorsed by the publisher.

## Abbreviations

MDD: Major depressive disorder
COVID-19: Coronavirus disease of 2019
AD: Alzheimer’s disease
SARS-CoV-2: Syndrome coronavirus 2
SNPs: Single nucleotide polymorphisms
GWAS: Genome wide association studies
MR: Mendelian randomization
IVs: Instrumental variables
ASSET: Association analysis based on subsets
FUMA: Functional mapping and annotation of genome-wide association studies
IVW: Inverse-variance weighted
GTEx: Genotype-Tissue Expression
