# Supplementary material for: Shared genetics and causal relationships between major depressive disorder and COVID-19 related traits: a large-scale genome-wide cross-trait meta-analysis
Source: Front Psychiatry. 2023 Jun 23;14:1144697. doi: 10.3389/fpsyt.2023.1144697 (PMC10328439; doi:10.3389/fpsyt.2023.1144697)
Supplement: Supplementary file 1 [file Data_Sheet_1.pdf]

Supplementary Material

1 Supplementary Figures and Tables

1.1 Supplementary Figures

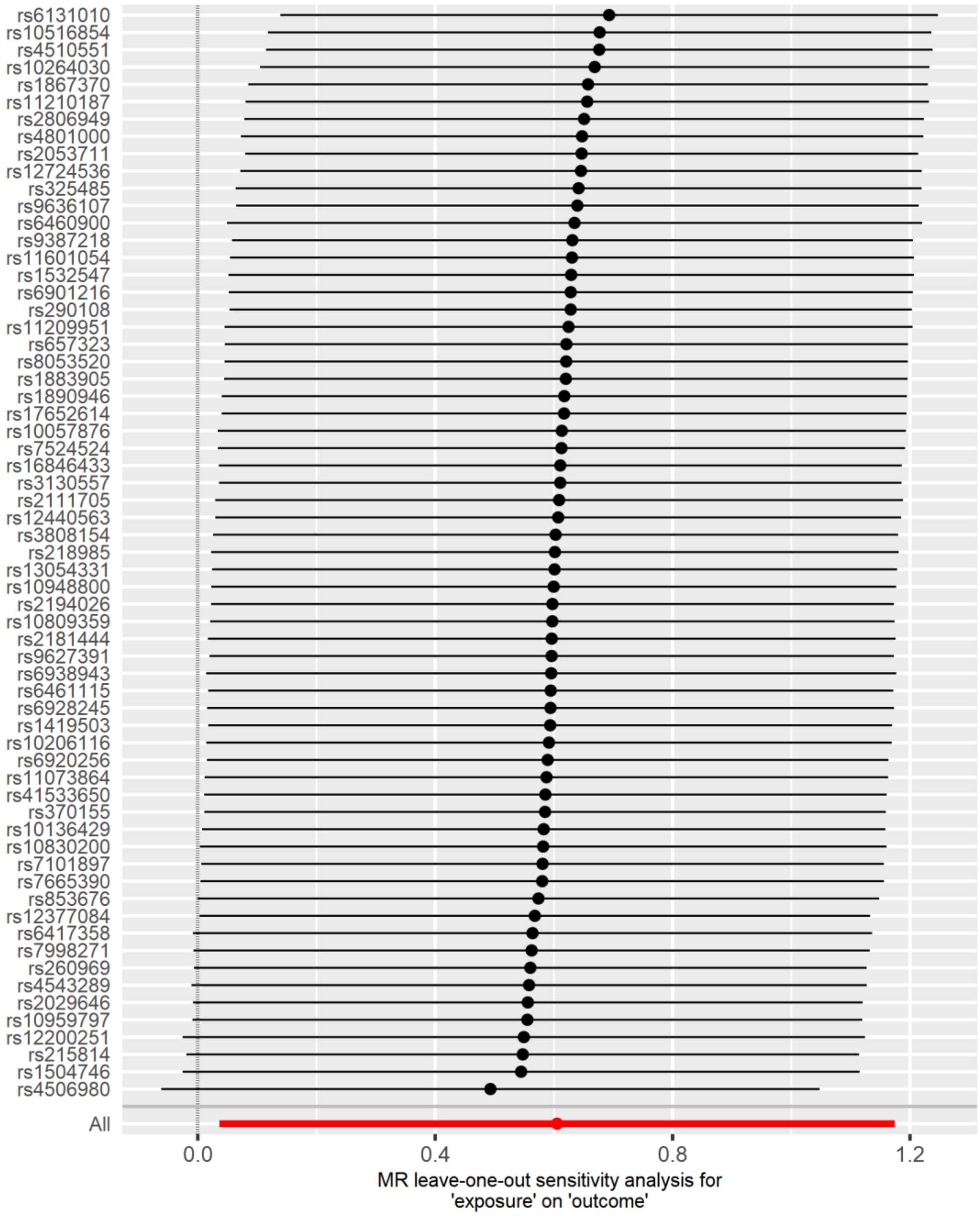

Supplementary Figure S1. Leave-one-out sensitivity analysis for the bidirectional Mendelian randomization analysis. (Direction: major depressive disorder → severe COVID-19)

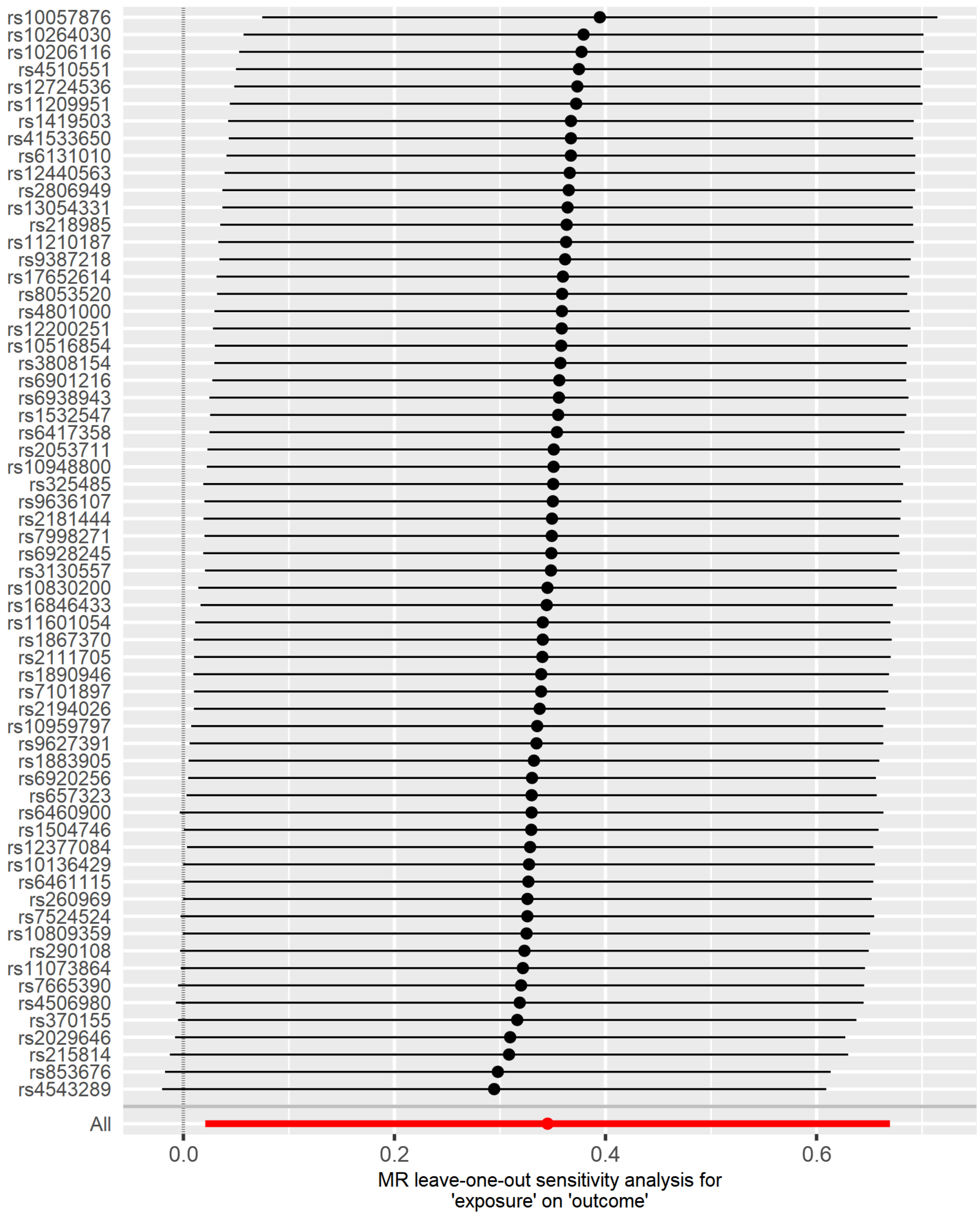

Supplementary Figure S2. Leave-one-out sensitivity analysis for the bidirectional Mendelian randomization analysis. (Direction: major depressive disorder → hospitalized COVID-19)

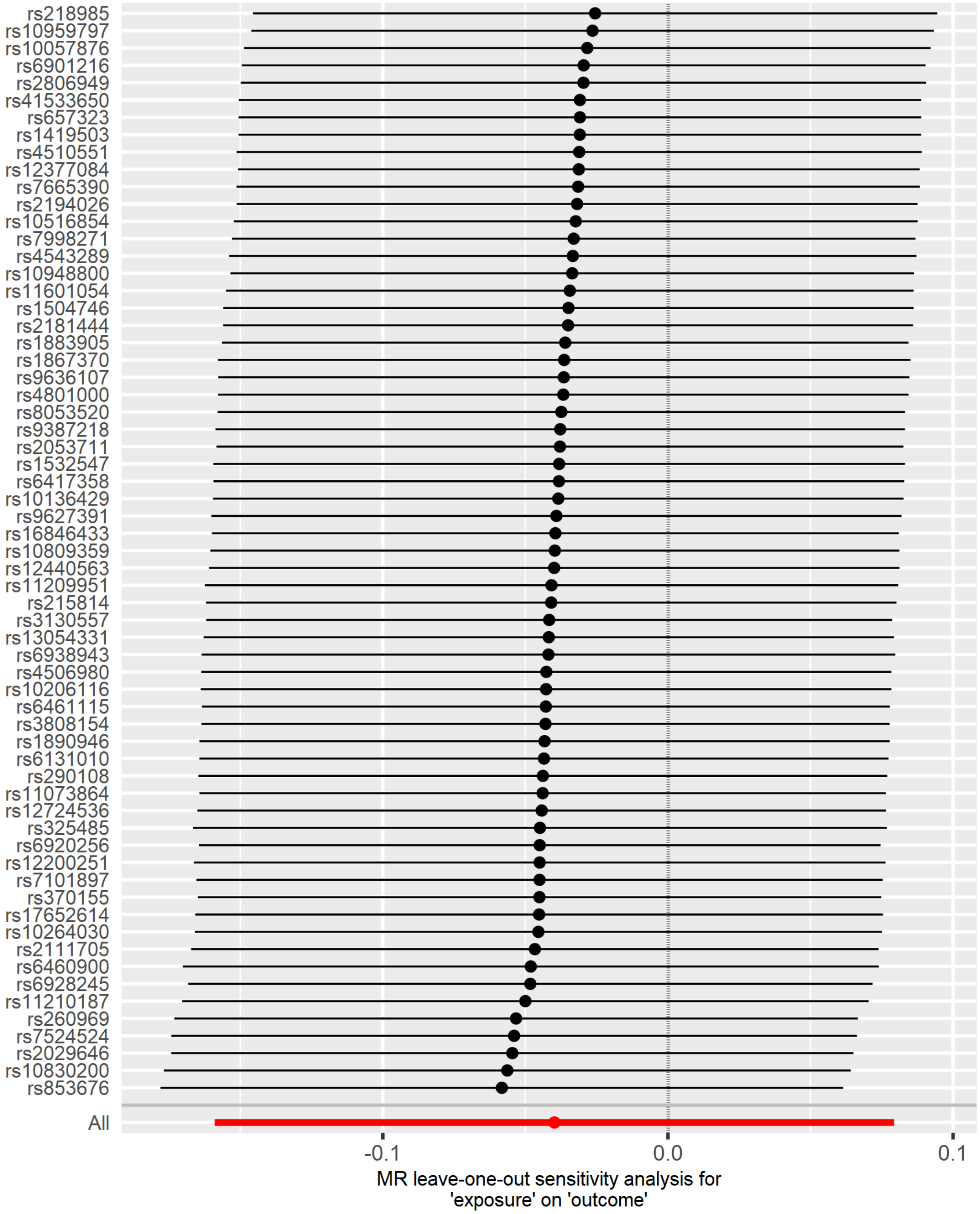

Supplementary Figure S3. Leave-one-out sensitivity analysis for the bidirectional Mendelian randomization analysis. (Direction: major depressive disorder → COVID-19 infection)

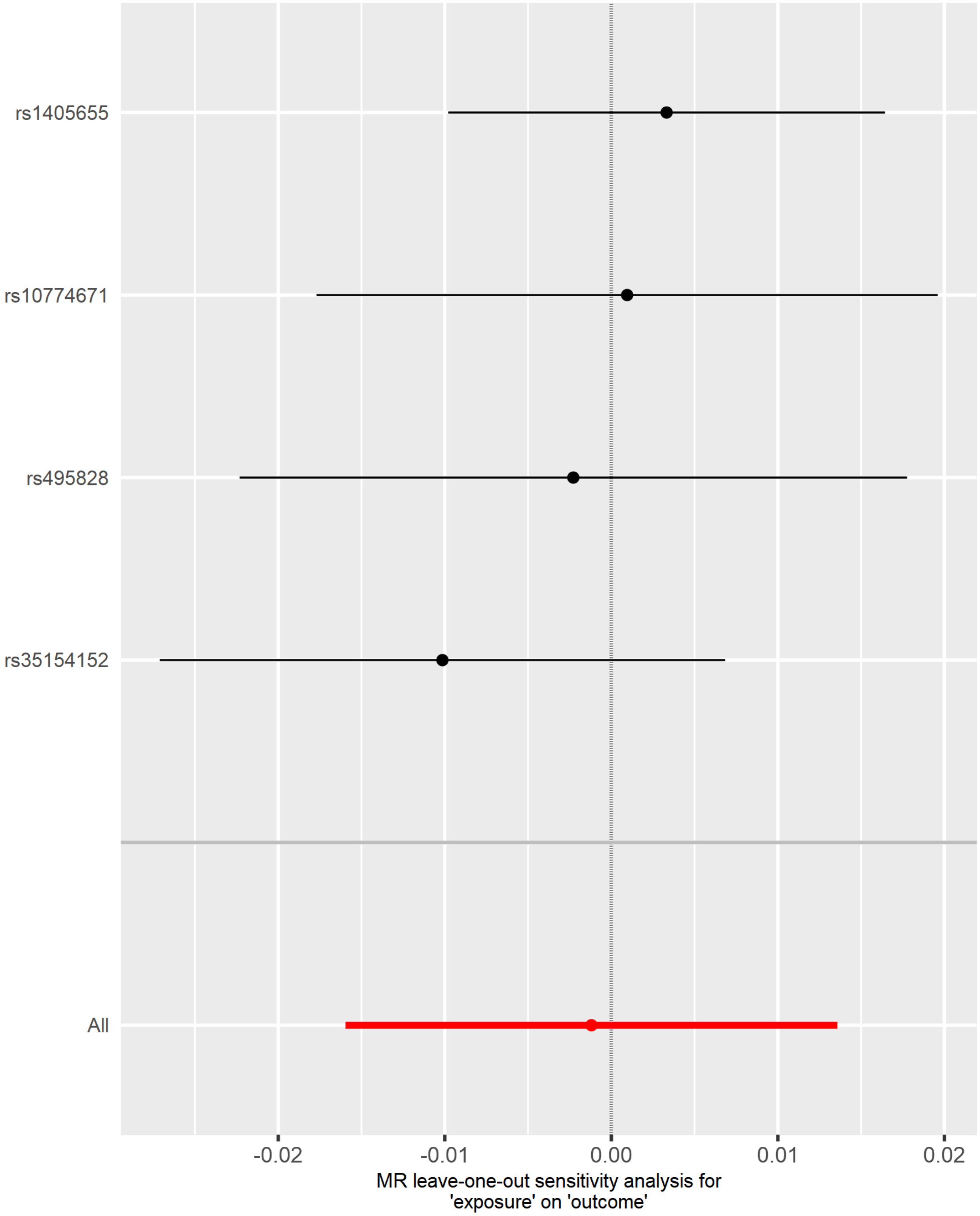

Supplementary Figure S4. Leave-one-out sensitivity analysis for the bidirectional Mendelian randomization analysis. (Direction: severe COVID-19 → major depressive disorder)

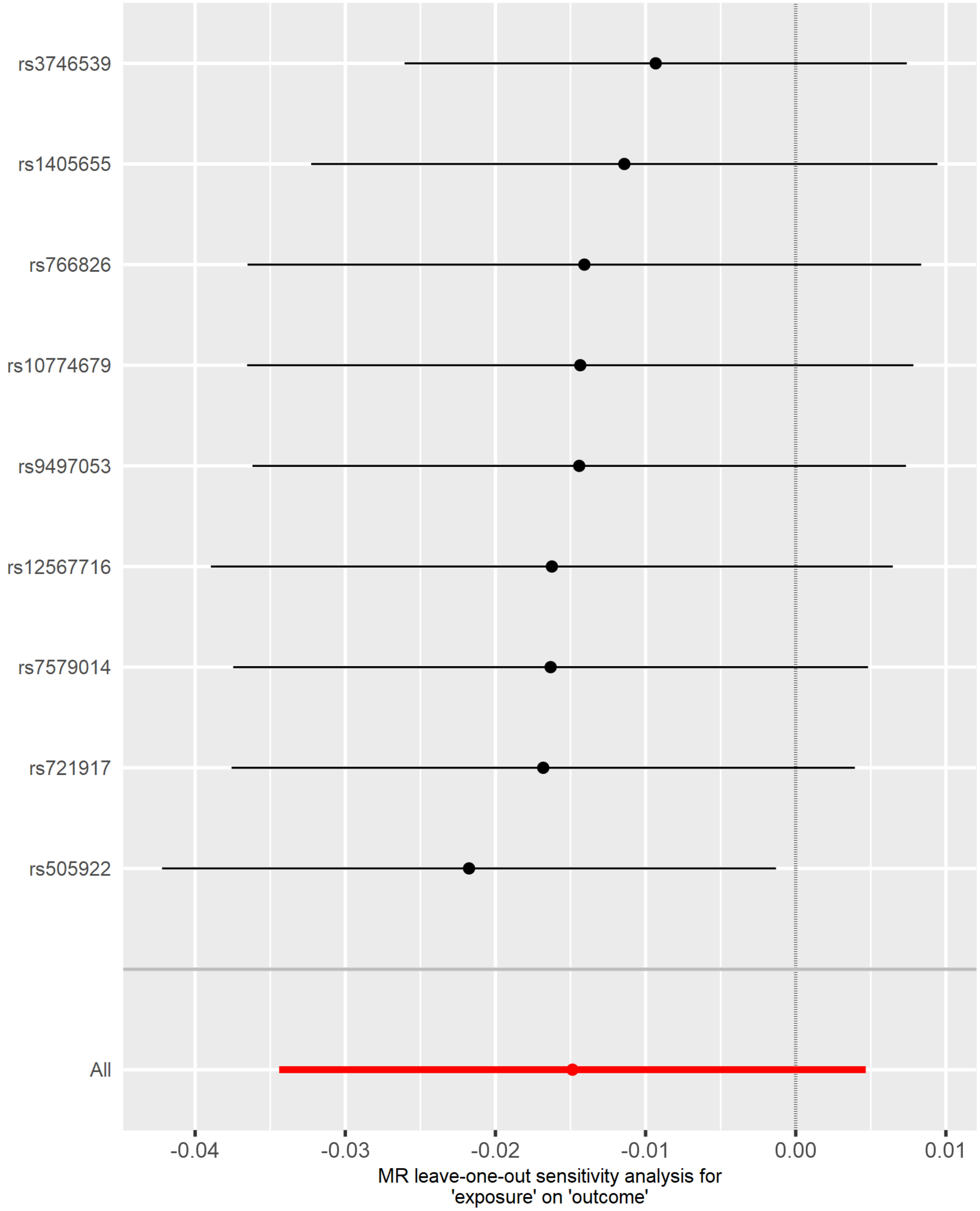

Supplementary Figure S5. Leave-one-out sensitivity analysis for the bidirectional Mendelian randomization analysis. (Direction: hospitalized COVID-19 → major depressive disorder)

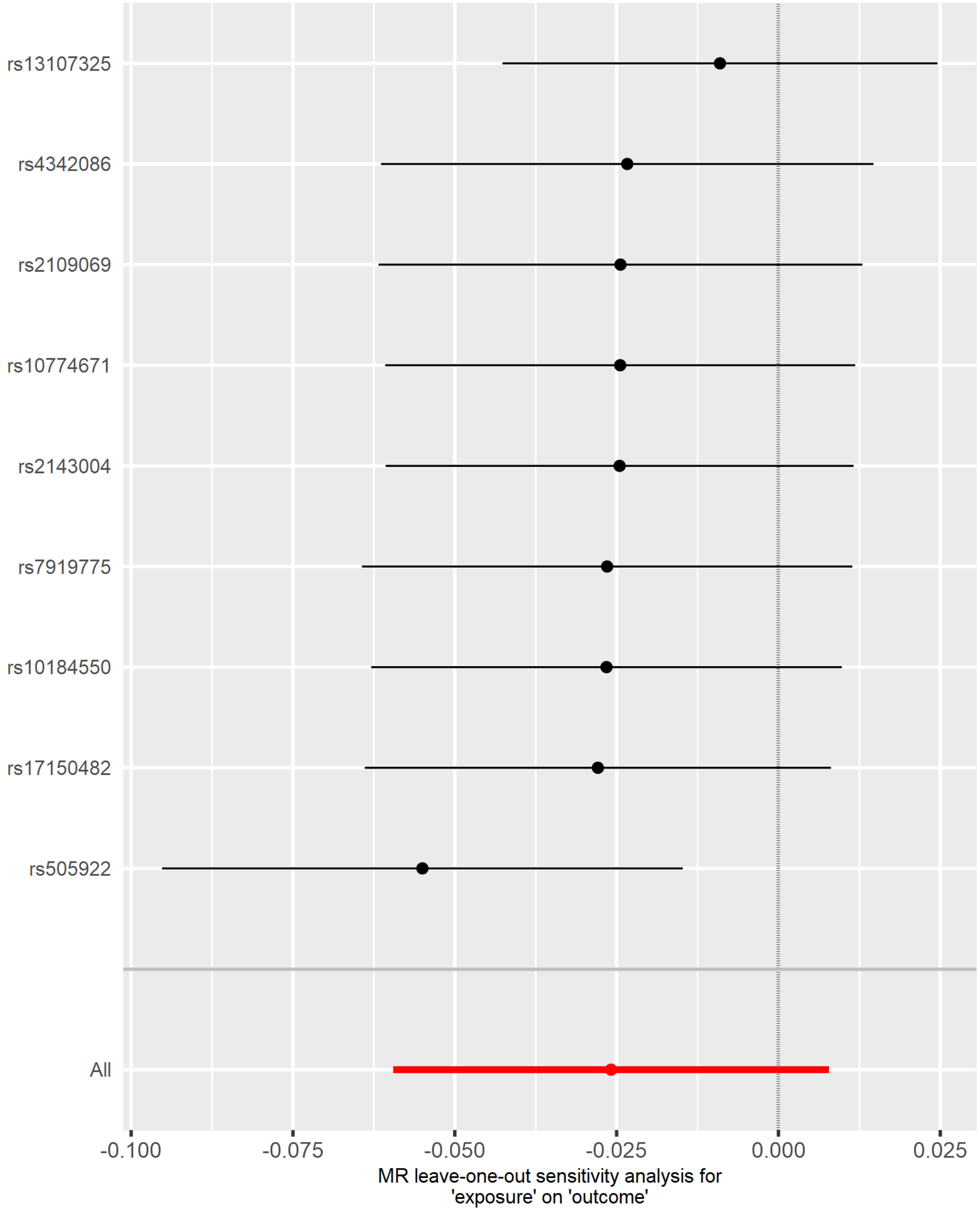

Supplementary Figure S6. Leave-one-out sensitivity analysis for the bidirectional Mendelian randomization analysis. (Direction: COVID-19 infection → major depressive disorder)

1.2 Supplementary Tables

Supplementary Table S1. Summary of GWAS data sets analyzed.

Phenotype	Cases	Controls	Ancestry	Sample size
MDD	65075	232552	European	297627
severe COVID-19	8779	1001875	European	1010654
hospitalized COVID-19	24274	2061529	European	2085803
COVID-19 infection	112612	2474079	European	2586691

Supplementary Table S2. Genome-wide significant genes by cross-trait meta-analysis associated with major depressive disorder and severe COVID-19.

SNP	chromosome	position	Chromosmoe Loci	A1	A2	P_+	P_-	P_{meta}	DIST	Genes within clumping region
rs2234358	3	45989044	3p21.31	G	T	2.46×10^{-11}	1.00	2.46×10^{-11}	0	*FYCO1:CXCR6*
rs4682799	3	45993606	3p21.31	C	T	1.24×10^{-10}	1.00	1.24×10^{-10}	0	*FYCO1*
rs17213127	3	45798226	3p21.31	T	C	2.02×10^{-10}	1.00	2.02×10^{-10}	0	*SLC6A20*
rs1396862	17	43902997	17q21.31	A	G	7.05×10^{-4}	6.90×10^{-8}	1.20×10^{-9}	0	*CRHR1*
rs17689824	17	43904397	17q21.31	T	C	7.15×10^{-4}	6.81×10^{-8}	1.21×10^{-9}	0	*CRHR1*
rs17689471	17	43892973	17q21.31	C	T	7.22×10^{-4}	6.79×10^{-8}	1.21×10^{-9}	0	*CRHR1:RP11-105N13.4*
rs17762769	17	43893403	17q21.31	A	G	6.99×10^{-4}	7.55×10^{-8}	1.30×10^{-9}	0	*CRHR1:RP11-105N13.4*
rs4566211	17	43895696	17q21.31	A	G	7.32×10^{-4}	7.37×10^{-8}	1.33×10^{-9}	0	*CRHR1*
rs16940665	17	43907896	17q21.31	C	T	7.31×10^{-4}	7.71×10^{-8}	1.39×10^{-9}	0	*CRHR1*
rs17689918	17	43910088	17q21.31	A	G	8.40×10^{-4}	6.75×10^{-8}	1.39×10^{-9}	0	*CRHR1*
rs17763086	17	43905481	17q21.31	G	T	7.20×10^{-4}	8.08×10^{-8}	1.43×10^{-9}	0	*CRHR1*
rs4277389	17	43895653	17q21.31	G	A	8.04×10^{-4}	7.36×10^{-8}	1.45×10^{-9}	0	*CRHR1*
rs17762954	17	43899786	17q21.31	T	C	7.64×10^{-4}	8.14×10^{-8}	1.52×10^{-9}	0	*CRHR1*
rs1876829	17	43911443	17q21.31	C	T	7.19×10^{-4}	8.97×10^{-8}	1.58×10^{-9}	0	*CRHR1*
rs8098405	18	50752373	18q21.2	G	A	2.04×10^{-7}	3.70×10^{-4}	1.84×10^{-9}	0	*DCC*
rs8099160	18	50752610	18q21.2	A	G	2.05×10^{-7}	3.71×10^{-4}	1.84×10^{-9}	0	*DCC*
rs17689882	17	43906828	17q21.31	A	G	7.61×10^{-4}	1.16×10^{-7}	2.14×10^{-9}	0	*CRHR1*
rs1557866	12	113405181	12q24.13	C	A	2.25×10^{-9}	1.00	2.25×10^{-9}	0	*RP1-71H24.1:OAS3*
rs10744791	12	113410316	12q24.13	A	G	2.28×10^{-9}	1.00	2.28×10^{-9}	0	*RP1-71H24.1:OAS3*
rs2285932	12	113386950	12q24.13	C	T	2.46×10^{-9}	1.00	2.46×10^{-9}	0	*RP1-71H24.1:OAS3*
rs1405655	19	50882619	19q13.33	C	T	3.11×10^{-9}	1.00	3.11×10^{-9}	0	*NR1H2*
rs8089828	18	50669725	18q21.2	C	T	5.31×10^{-4}	2.53×10^{-7}	3.19×10^{-9}	0	*DCC*
rs4414555	18	50643812	18q21.2	C	T	3.17×10^{-7}	4.93×10^{-4}	3.68×10^{-9}	0	*DCC*
rs8072451	17	43893716	17q21.31	T	C	6.89×10^{-4}	3.10×10^{-7}	4.96×10^{-9}	0	*CRHR1:RP11-105N13.4*
rs676314	19	50865535	19q13.33	G	A	6.98×10^{-9}	1.00	6.98×10^{-9}	0	*NR1H2:NAPSA*
rs2300371	21	34632241	21q22.11	T	C	7.88×10^{-9}	1.00	7.88×10^{-9}	0	*IFNAR2:AP000295.9*
rs17488727	18	50760933	18q21.2	C	A	1.31×10^{-7}	3.33×10^{-3}	9.83×10^{-9}	0	*DCC*

rs4506980	18	50597654	18q21.2	A	G	4.58×10^{-7}	1.22×10^{-3}	1.25×10^{-8}	0	*DCC*
rs17487256	18	50718314	18q21.2	T	C	7.93×10^{-8}	7.28×10^{-3}	1.29×10^{-8}	0	*DCC*
rs3794923	18	50727518	18q21.2	T	C	7.42×10^{-8}	8.16×10^{-3}	1.35×10^{-8}	0	*DCC*
rs1431196	18	50832102	18q21.2	G	A	8.27×10^{-8}	8.41×10^{-3}	1.54×10^{-8}	0	*DCC*
rs1274517	19	50868754	19q13.33	G	A	1.58×10^{-8}	1.00	1.58×10^{-8}	0	*NR1H2:NAPSA*
rs4129439	18	50589083	18q21.2	C	T	4.65×10^{-7}	1.60×10^{-3}	1.63×10^{-8}	0	*DCC*
rs4468701	18	50611280	18q21.2	C	T	5.35×10^{-7}	2.37×10^{-3}	2.72×10^{-8}	0	*DCC*
rs4459619	18	50620708	18q21.2	C	T	3.80×10^{-7}	3.38×10^{-3}	2.76×10^{-8}	0	*DCC*
rs4321259	18	50623640	18q21.2	A	G	3.08×10^{-7}	4.21×10^{-3}	2.78×10^{-8}	0	*DCC*
rs7232543	18	50718757	18q21.2	G	A	2.37×10^{-7}	5.54×10^{-3}	2.82×10^{-8}	0	*DCC*
rs1274514	19	50871147	19q13.33	G	A	2.88×10^{-8}	1.00	2.88×10^{-8}	0	*NR1H2:CTB-191K22.6*
rs1546079	3	46150119	3p21.31	T	C	2.95×10^{-8}	1.00	2.95×10^{-8}	33730	*FLT1P1*
rs4291980	18	50617175	18q21.2	G	T	2.35×10^{-7}	6.01×10^{-3}	3.02×10^{-8}	0	*DCC*
rs199442	17	44820122	17q21.31	A	G	7.90×10^{-3}	1.87×10^{-7}	3.15×10^{-8}	0	*NSF*
rs1431181	18	50779412	18q21.2	A	G	1.44×10^{-7}	1.04×10^{-2}	3.20×10^{-8}	0	*DCC*
rs3821884	3	46016599	3p21.31	G	T	3.34×10^{-8}	1.00	3.34×10^{-8}	0	*FYCO1*
rs199444	17	44818276	17q21.31	C	T	8.02×10^{-3}	2.35×10^{-7}	3.97×10^{-8}	0	*NSF*
rs17411061	18	50795113	18q21.2	T	C	1.64×10^{-7}	1.16×10^{-2}	4.01×10^{-8}	0	*DCC*
rs199535	17	44822662	17q21.31	G	A	1.32×10^{-2}	1.49×10^{-7}	4.14×10^{-8}	0	*NSF*

SNP: single nucleotide polymorphism; A1: effect allele; A2: other allele; P_+: P-value for positive direction; P_-: P-value for negative direction; P_{meta}: P-value for cross-trait meta-analysis. DIST: Distance to the nearest gene. SNPs which are locating in the gene body or 1kb up- or down-stream of transcription start site or transcription end site have 0.

Supplementary Table S3. Genome-wide significant genes by cross-trait meta-analysis associated with major depressive disorder and hospitalized COVID-19.

SNP	chromosome	position	Chromosmoe Loci	A1	A2	P_+	P_-	P_{meta}	DIST	Genes within clumping region
rs17689471	17	43892973	17q21.31	C	T	7.22×10^{-4}	6.46×10^{-11}	1.48×10^{-12}	0	*CRHR1:RP11-105N13.4*
rs17689824	17	43904397	17q21.31	T	C	7.15×10^{-4}	7.28×10^{-11}	1.64×10^{-12}	0	*CRHR1*
rs1396862	17	43902997	17q21.31	A	G	7.05×10^{-4}	7.38×10^{-11}	1.64×10^{-12}	0	*CRHR1*
rs16940665	17	43907896	17q21.31	C	T	7.31×10^{-4}	7.77×10^{-11}	1.79×10^{-12}	0	*CRHR1*
rs17763086	17	43905481	17q21.31	G	T	7.20×10^{-4}	8.00×10^{-11}	1.81×10^{-12}	0	*CRHR1*
rs17762769	17	43893403	17q21.31	A	G	6.99×10^{-4}	8.60×10^{-11}	1.89×10^{-12}	0	*CRHR1:RP11-105N13.4*
rs4566211	17	43895696	17q21.31	A	G	7.32×10^{-4}	8.81×10^{-11}	2.02×10^{-12}	0	*CRHR1*
rs1876829	17	43911443	17q21.31	C	T	7.19×10^{-4}	9.15×10^{-11}	2.06×10^{-12}	0	*CRHR1*
rs17762954	17	43899786	17q21.31	T	C	7.64×10^{-4}	9.02×10^{-11}	2.16×10^{-12}	0	*CRHR1*
rs4277389	17	43895653	17q21.31	G	A	8.04×10^{-4}	9.04×10^{-11}	2.27×10^{-12}	0	*CRHR1*
rs17689882	17	43906828	17q21.31	A	G	7.61×10^{-4}	9.91×10^{-11}	2.35×10^{-12}	0	*CRHR1*
rs17689918	17	43910088	17q21.31	A	G	8.40×10^{-4}	9.61×10^{-11}	2.51×10^{-12}	0	*CRHR1*
rs8072451	17	43893716	17q21.31	T	C	6.89×10^{-4}	9.00×10^{-10}	1.81×10^{-11}	0	*CRHR1:RP11-105N13.4*
rs853676	6	28299687	6p22.1	T	C	8.69×10^{-3}	1.19×10^{-9}	2.71×10^{-10}	0	*ZSCAN31*
rs199457	17	44795469	17q21.31	T	C	1.01×10^{-2}	1.79×10^{-9}	4.66×10^{-10}	0	*NSF*
rs199456	17	44797919	17q21.31	T	C	1.13×10^{-2}	2.51×10^{-9}	7.16×10^{-10}	0	*NSF*
rs199451	17	44801784	17q21.31	A	G	1.05×10^{-2}	3.23×10^{-9}	8.53×10^{-10}	0	*NSF*
rs199448	17	44809001	17q21.31	G	A	1.06×10^{-2}	3.25×10^{-9}	8.62×10^{-10}	0	*NSF*
rs199535	17	44822662	17q21.31	G	A	1.32×10^{-2}	2.78×10^{-9}	9.15×10^{-10}	0	*NSF*
rs169201	17	44790203	17q21.31	G	A	9.45×10^{-3}	3.98×10^{-9}	9.40×10^{-10}	0	*NSF*
rs199533	17	44828931	17q21.31	A	G	1.51×10^{-2}	2.76×10^{-9}	1.04×10^{-9}	0	*NSF*
rs853681	6	28296650	6p22.1	A	C	1.47×10^{-2}	3.07×10^{-9}	1.12×10^{-9}	0	*ZSCAN31*
rs853679	6	28296863	6p22.1	A	C	1.49×10^{-2}	3.09×10^{-9}	1.14×10^{-9}	0	*ZSCAN31*
rs199534	17	44824213	17q21.31	G	T	1.33×10^{-2}	3.55×10^{-9}	1.16×10^{-9}	0	*NSF*
rs199439	17	44793503	17q21.31	G	A	9.90×10^{-3}	6.91×10^{-9}	1.67×10^{-9}	0	*NSF*
rs853685	6	28288785		T	C	2.16×10^{-2}	3.44×10^{-9}	1.80×10^{-9}	1155	*RP5-874C20.6*
rs7118388	11	34454147		G	A	3.39×10^{-9}	1.00	3.39×10^{-9}		

rs1419183	6	28242794		C	A	3.91×10^{-2}	4.81×10^{-9}	4.40×10^{-9}	0	*RP5-874C20.3*
rs10456362	6	28221816	6p22.1	G	A	1.22×10^{-8}	1.73×10^{-2}	4.90×10^{-9}	0	*ZKSCAN4*
rs11088268	21	35363759	21q22.11	G	T	9.90×10^{-9}	3.19×10^{-2}	7.23×10^{-9}	13766	*LINC00649*
rs199443	17	44819565	17q21.31	T	C	1.28×10^{-2}	2.55×10^{-8}	7.45×10^{-9}	0	*NSF*
rs1679709	6	28228342	6p22.1	G	A	7.38×10^{-9}	4.46×10^{-2}	7.52×10^{-9}	0	*NKAPL*
rs1778508	6	28229881	6p22.1	T	C	8.59×10^{-9}	4.01×10^{-2}	7.85×10^{-9}	1144	*NKAPL*
rs11965538	6	28239915		A	G	4.21×10^{-2}	9.48×10^{-9}	9.03×10^{-9}	0	*RP5-874C20.3*
rs6901575	6	28250984	6p22.1	A	G	4.40×10^{-2}	9.59×10^{-9}	9.54×10^{-9}	0	*PGBD1*
rs2799077	6	28234597		T	C	4.31×10^{-2}	1.57×10^{-8}	1.49×10^{-8}	190	*RP5-874C20.3*
rs17720293	6	28214698	6p22.1	T	C	4.13×10^{-2}	3.23×10^{-8}	2.86×10^{-8}	0	*ZKSCAN4*
rs199513	17	44856932	17q21.31-q21.32	G	A	1.10×10^{-7}	1.39×10^{-2}	3.25×10^{-8}	0	*WNT3*
rs3746539	20	40702071		A	G	4.43×10^{-8}	1.00	4.43×10^{-8}		

SNP: single nucleotide polymorphism; A1: effect allele; A2: other allele; P_+: *P*-value for positive direction; P_-: *P*-value for negative direction; P_{meta}: *P*-value for cross-trait meta-analysis. DIST: Distance to the nearest gene. SNPs which are locating in the gene body or 1kb up- or down-stream of transcription start site or transcription end site have 0.

Supplementary Table S4. Genome-wide significant genes by cross-trait meta-analysis associated with major depressive disorder and COVID-19 infection.

SNP	chromosome	position	Chromosmoe Loci	A1	A2	P_{+}	P_{-}	P_{meta}	DIST	Genes within clumping region
rs853676	6	28299687	6p22.1	T	C	8.79×10^{-3}	1.19×10^{-9}	2.74×10^{-10}	0	*ZSCAN31*
rs4683856	3	101534326		A	G	5.64×10^{-10}	1.00	5.64×10^{-10}		
rs853679	6	28296863	6p22.1	A	C	1.23×10^{-2}	3.09×10^{-9}	9.53×10^{-10}	0	*ZSCAN31*
rs853681	6	28296650	6p22.1	A	C	1.26×10^{-2}	3.07×10^{-9}	9.65×10^{-10}	0	*ZSCAN31*
rs853685	6	28288785		T	C	1.35×10^{-2}	3.44×10^{-9}	1.15×10^{-9}	1155	*RP5-874C20.6*
rs6797522	3	101543603		A	G	1.52×10^{-9}	1.00	1.52×10^{-9}		
rs1419183	6	28242794		C	A	1.99×10^{-2}	4.81×10^{-9}	2.30×10^{-9}	0	*RP5-874C20.3*
rs1778508	6	28229881	6p22.1	T	C	8.59×10^{-9}	2.16×10^{-2}	4.35×10^{-9}	1144	*NKAPL*
rs2303473	3	101404813		G	A	4.39×10^{-9}	1.00	4.39×10^{-9}		
rs1679709	6	28228342	6p22.1	G	A	7.38×10^{-9}	2.71×10^{-2}	4.66×10^{-9}	0	*NKAPL*
rs11965538	6	28239915		A	G	2.13×10^{-2}	9.48×10^{-9}	4.72×10^{-9}	0	*RP5-874C20.3*
rs6791696	3	101409096		G	A	4.72×10^{-9}	1.00	4.72×10^{-9}		
rs6901575	6	28250984	6p22.1	A	G	2.30×10^{-2}	9.59×10^{-9}	5.14×10^{-9}	0	*PGBD1*
rs10456362	6	28221816	6p22.1	G	A	1.22×10^{-8}	1.86×10^{-2}	5.26×10^{-9}	0	*ZKSCAN4*
rs2081577	3	101356219		A	G	6.50×10^{-9}	1.00	6.50×10^{-9}		
rs10936715	3	101345965		G	A	1.00	6.67×10^{-9}	6.67×10^{-9}		
rs6441620	3	101349644		A	C	1.00	7.20×10^{-9}	7.20×10^{-9}		
rs2799077	6	28234597		T	C	2.20×10^{-2}	1.57×10^{-8}	7.87×10^{-9}	190	*RP5-874C20.3*
rs4683939	3	101362589		G	A	8.14×10^{-9}	1.00	8.14×10^{-9}		
rs11916199	3	101507015		T	G	1.01×10^{-8}	1.00	1.01×10^{-8}		
rs2466367	3	101501827		C	T	1.00	1.39×10^{-8}	1.39×10^{-8}		
rs677483	11	88701202	11q14.2-q14.3	G	A	2.67×10^{-2}	4.44×10^{-8}	2.55×10^{-8}	0	*GRM5*
rs12638134	3	101334609		T	G	1.00	2.97×10^{-8}	2.97×10^{-8}		
rs13099833	3	100878496	3q12.2	G	A	1.91×10^{-3}	9.67×10^{-7}	3.91×10^{-8}	3441	*ACTR3P3*

SNP: single nucleotide polymorphism; A1: effect allele; A2: other allele; P_+: *P*-value for positive direction; P_-: *P*-value for negative direction; P_{meta}: *P*-value for cross-trait meta-analysis. DIST: Distance to the nearest gene. SNPs which are locating in the gene body or 1kb up- or down-stream of transcription start site or transcription end site have 0.

frontiers

Supplementary Table S5. Instrumental variables used in bidirectional Mendelian randomization analysis.

Direction	chromosome	position	Effect allele	Other allele	beta	SE	*p*-value	SNP
MDD ↓ severe COVID-19	1	37169665	C	T	-7.54×10^{-3}	1.91×10^{-2}	6.94×10^{-1}	rs218985
	1	37667516	T	C	-3.96×10^{-2}	2.11×10^{-2}	6.08×10^{-2}	rs215814
	1	39551621	C	T	-4.29×10^{-2}	2.48×10^{-2}	8.29×10^{-2}	rs260969
	1	52342427	T	C	1.84×10^{-3}	2.11×10^{-2}	9.30×10^{-1}	rs1890946
	1	72837490	C	T	2.07×10^{-3}	1.98×10^{-2}	9.17×10^{-1}	rs11209951
	1	73446704	G	A	1.35×10^{-2}	1.81×10^{-2}	4.56×10^{-1}	rs12724536
	1	73715233	T	C	1.35×10^{-2}	1.82×10^{-2}	4.56×10^{-1}	rs11210187
	1	74097006	T	C	2.28×10^{-3}	2.00×10^{-2}	9.09×10^{-1}	rs7524524
	1	173820593	G	A	-1.46×10^{-3}	2.99×10^{-2}	9.61×10^{-1}	rs16846433
	1	177407630	A	G	6.17×10^{-3}	2.44×10^{-2}	8.01×10^{-1}	rs1883905
	2	15450646	T	C	1.37×10^{-2}	2.10×10^{-2}	5.15×10^{-1}	rs10206116
	3	7371919	A	G	2.00×10^{-3}	2.06×10^{-2}	9.23×10^{-1}	rs17652614
	4	15577738	G	A	-1.93×10^{-2}	1.98×10^{-2}	3.31×10^{-1}	rs7665390
	4	90916437	C	T	3.42×10^{-2}	1.92×10^{-2}	7.43×10^{-2}	rs10516854
	4	176869252	G	A	-2.02×10^{-2}	2.29×10^{-2}	3.79×10^{-1}	rs41533650
	5	61487083	C	T	-2.57×10^{-3}	1.98×10^{-2}	8.97×10^{-1}	rs10057876
	5	87801273	G	T	-1.46×10^{-2}	2.90×10^{-2}	6.14×10^{-1}	rs2194026
	5	103729262	C	T	3.56×10^{-2}	2.15×10^{-2}	9.80×10^{-2}	rs4510551
	5	103995368	A	G	-1.00×10^{-2}	2.13×10^{-2}	6.39×10^{-1}	rs325485
	5	164484948	T	G	-3.51×10^{-2}	2.12×10^{-2}	9.82×10^{-2}	rs4543289
	6	13625413	A	G	9.88×10^{-3}	2.36×10^{-2}	6.75×10^{-1}	rs10948800
	6	26537801	G	A	2.35×10^{-2}	2.88×10^{-2}	4.14×10^{-1}	rs6920256
	6	27376910	G	A	-1.07×10^{-2}	1.91×10^{-2}	5.73×10^{-1}	rs6938943
	6	27782031	T	G	2.79×10^{-2}	3.01×10^{-2}	3.54×10^{-1}	rs370155
	6	28299687	C	T	3.06×10^{-2}	2.61×10^{-2}	2.40×10^{-1}	rs853676
	6	31094703	C	T	-2.81×10^{-3}	3.34×10^{-2}	9.33×10^{-1}	rs3130557
	6	33807091	T	C	-7.03×10^{-3}	1.92×10^{-2}	7.14×10^{-1}	rs6901216
	6	97734271	G	A	-1.48×10^{-2}	2.38×10^{-2}	5.35×10^{-1}	rs9387218
	6	100674018	A	G	-1.08×10^{-2}	1.87×10^{-2}	5.64×10^{-1}	rs6928245
	6	105382415	G	A	-2.82×10^{-2}	1.82×10^{-2}	1.22×10^{-1}	rs12200251

7	2103668	A	G	1.17×10^{-2}	2.04×10^{-2}	5.66×10^{-1}	rs6461115
7	4204008	A	G	7.23×10^{-3}	2.40×10^{-2}	7.63×10^{-1}	rs657323
7	12253088	A	G	4.28×10^{-4}	1.79×10^{-2}	9.81×10^{-1}	rs6460900
7	82735751	G	A	2.84×10^{-2}	1.89×10^{-2}	1.32×10^{-1}	rs10264030
7	125867303	A	C	-1.55×10^{-2}	2.53×10^{-2}	5.39×10^{-1}	rs1419503
7	126331987	A	G	-7.32×10^{-3}	2.30×10^{-2}	7.51×10^{-1}	rs3808154
9	11145717	T	C	1.26×10^{-2}	2.55×10^{-2}	6.21×10^{-1}	rs10809359
9	11431990	G	A	4.56×10^{-2}	2.40×10^{-2}	5.73×10^{-2}	rs10959797
9	11781428	C	A	4.79×10^{-2}	2.75×10^{-2}	8.17×10^{-2}	rs12377084
9	37223862	A	G	-4.59×10^{-2}	2.41×10^{-2}	5.73×10^{-2}	rs2029646
10	10842639	G	A	1.01×10^{-2}	1.91×10^{-2}	5.96×10^{-1}	rs2181444
11	31010962	C	T	-1.04×10^{-2}	2.16×10^{-2}	6.29×10^{-1}	rs290108
11	88475938	A	G	6.36×10^{-3}	2.09×10^{-2}	7.61×10^{-1}	rs1532547
11	88745439	T	C	1.75×10^{-2}	2.01×10^{-2}	3.83×10^{-1}	rs10830200
11	89007922	C	T	-2.01×10^{-2}	2.03×10^{-2}	3.24×10^{-1}	rs7101897
11	113336172	G	A	-8.01×10^{-3}	1.96×10^{-2}	6.83×10^{-1}	rs11601054
13	31862334	C	A	-3.33×10^{-2}	2.14×10^{-2}	1.20×10^{-1}	rs7998271
13	53646138	A	G	-1.82×10^{-2}	2.04×10^{-2}	3.71×10^{-1}	rs2806949
13	53898419	T	G	2.12×10^{-2}	2.24×10^{-2}	3.43×10^{-1}	rs1867370
13	69741897	A	G	3.26×10^{-2}	2.63×10^{-2}	2.15×10^{-1}	rs2053711
14	75261641	G	A	-4.03×10^{-3}	1.80×10^{-2}	8.22×10^{-1}	rs2111705
14	99706474	G	A	1.98×10^{-2}	2.15×10^{-2}	3.57×10^{-1}	rs10136429
15	74060297	G	A	4.15×10^{-3}	2.02×10^{-2}	8.37×10^{-1}	rs12440563
15	89989514	C	T	-1.77×10^{-2}	2.22×10^{-2}	4.25×10^{-1}	rs11073864
16	13756549	C	A	6.89×10^{-3}	2.43×10^{-2}	7.77×10^{-1}	rs8053520
18	50597654	G	A	-6.28×10^{-2}	1.94×10^{-2}	1.22×10^{-3}	rs4506980
18	50858019	T	C	-3.42×10^{-2}	1.94×10^{-2}	7.83×10^{-2}	rs1504746
18	53200117	A	G	1.24×10^{-2}	2.02×10^{-2}	5.39×10^{-1}	rs9636107
18	53456943	A	G	1.31×10^{-2}	1.86×10^{-2}	4.81×10^{-1}	rs4801000
20	44724305	A	G	-4.88×10^{-2}	2.22×10^{-2}	2.75×10^{-2}	rs6131010
20	59002996	C	A	2.91×10^{-2}	2.04×10^{-2}	1.54×10^{-1}	rs6417358
22	23407063	A	G	-9.34×10^{-3}	2.49×10^{-2}	7.08×10^{-1}	rs13054331
22	46447097	C	T	1.24×10^{-2}	2.34×10^{-2}	5.96×10^{-1}	rs9627391

MDD ↓ hospitalized COVID-19	1	37169665	C	T	6.09×10^{-3}	1.14×10^{-2}	5.92×10^{-1}	rs218985
	1	37667516	T	C	-2.52×10^{-2}	1.21×10^{-2}	3.79×10^{-2}	rs215814
	1	39551621	C	T	-1.73×10^{-2}	1.33×10^{-2}	1.92×10^{-1}	rs260969
	1	52342427	T	C	-6.46×10^{-3}	1.13×10^{-2}	5.69×10^{-1}	rs1890946
	1	72837490	C	T	8.10×10^{-3}	1.12×10^{-2}	4.68×10^{-1}	rs11209951
	1	73446704	G	A	1.18×10^{-2}	1.09×10^{-2}	2.79×10^{-1}	rs12724536
	1	73715233	T	C	4.20×10^{-3}	1.10×10^{-2}	7.02×10^{-1}	rs11210187
	1	74097006	T	C	1.27×10^{-2}	1.11×10^{-2}	2.55×10^{-1}	rs7524524
	1	173820593	G	A	4.28×10^{-3}	1.80×10^{-2}	8.12×10^{-1}	rs16846433
	1	177407630	A	G	-1.46×10^{-2}	1.45×10^{-2}	3.15×10^{-1}	rs1883905
	2	15450646	T	C	-1.34×10^{-2}	1.12×10^{-2}	2.32×10^{-1}	rs10206116
	3	7371919	A	G	5.13×10^{-3}	1.12×10^{-2}	6.46×10^{-1}	rs17652614
	4	15577738	G	A	-1.85×10^{-2}	1.18×10^{-2}	1.17×10^{-1}	rs7665390
	4	90916437	C	T	4.62×10^{-3}	1.12×10^{-2}	6.81×10^{-1}	rs10516854
	4	176869252	G	A	1.52×10^{-2}	1.36×10^{-2}	2.62×10^{-1}	rs41533650
	5	61487083	C	T	1.93×10^{-2}	1.12×10^{-2}	8.44×10^{-2}	rs10057876
	5	87801273	G	T	-1.15×10^{-2}	1.60×10^{-2}	4.72×10^{-1}	rs2194026
	5	103729262	C	T	1.32×10^{-2}	1.20×10^{-2}	2.69×10^{-1}	rs4510551
	5	103995368	A	G	1.90×10^{-3}	1.14×10^{-2}	8.68×10^{-1}	rs325485
	5	164484948	T	G	-3.06×10^{-2}	1.12×10^{-2}	6.33×10^{-3}	rs4543289
	6	13625413	A	G	-1.53×10^{-3}	1.43×10^{-2}	9.15×10^{-1}	rs10948800
	6	26537801	G	A	1.94×10^{-2}	1.61×10^{-2}	2.27×10^{-1}	rs6920256
	6	27376910	G	A	3.87×10^{-4}	1.08×10^{-2}	9.72×10^{-1}	rs6938943
	6	27782031	T	G	3.19×10^{-2}	1.64×10^{-2}	5.19×10^{-2}	rs370155
	6	28299687	C	T	3.79×10^{-2}	1.44×10^{-2}	8.69×10^{-3}	rs853676
	6	31094703	C	T	-1.68×10^{-3}	1.88×10^{-2}	9.29×10^{-1}	rs3130557
	6	33807091	T	C	-3.24×10^{-3}	1.12×10^{-2}	7.73×10^{-1}	rs6901216
	6	97734271	G	A	-7.35×10^{-3}	1.22×10^{-2}	5.45×10^{-1}	rs9387218
	6	100674018	A	G	-1.38×10^{-3}	1.10×10^{-2}	9.00×10^{-1}	rs6928245
	6	105382415	G	A	1.92×10^{-3}	1.07×10^{-2}	8.58×10^{-1}	rs12200251
	7	2103668	A	G	1.47×10^{-2}	1.19×10^{-2}	2.19×10^{-1}	rs6461115

7	4204008	A	G	-1.48×10^{-2}	1.33×10^{-2}	2.68×10^{-1}	rs657323
7	12253088	A	G	-9.42×10^{-3}	1.06×10^{-2}	3.73×10^{-1}	rs6460900
7	82735751	G	A	1.57×10^{-2}	1.09×10^{-2}	1.49×10^{-1}	rs10264030
7	125867303	A	C	1.49×10^{-2}	1.42×10^{-2}	2.94×10^{-1}	rs1419503
7	126331987	A	G	5.74×10^{-3}	1.32×10^{-2}	6.63×10^{-1}	rs3808154
9	11145717	T	C	2.03×10^{-2}	1.46×10^{-2}	1.65×10^{-1}	rs10809359
9	11431990	G	A	1.15×10^{-2}	1.40×10^{-2}	4.12×10^{-1}	rs10959797
9	11781428	C	A	1.98×10^{-2}	1.47×10^{-2}	1.80×10^{-1}	rs12377084
9	37223862	A	G	-3.18×10^{-2}	1.36×10^{-2}	1.93×10^{-2}	rs2029646
10	10842639	G	A	1.34×10^{-3}	1.13×10^{-2}	9.06×10^{-1}	rs2181444
11	31010962	C	T	1.59×10^{-2}	1.14×10^{-2}	1.65×10^{-1}	rs290108
11	88475938	A	G	1.51×10^{-3}	1.16×10^{-2}	8.97×10^{-1}	rs1532547
11	88745439	T	C	3.71×10^{-3}	1.12×10^{-2}	7.39×10^{-1}	rs10830200
11	89007922	C	T	-6.79×10^{-3}	1.17×10^{-2}	5.61×10^{-1}	rs7101897
11	113336172	G	A	5.78×10^{-3}	1.16×10^{-2}	6.19×10^{-1}	rs11601054
13	31862334	C	A	-4.00×10^{-4}	1.23×10^{-2}	9.74×10^{-1}	rs7998271
13	53646138	A	G	-6.58×10^{-3}	1.12×10^{-2}	5.57×10^{-1}	rs2806949
13	53898419	T	G	-6.30×10^{-3}	1.23×10^{-2}	6.07×10^{-1}	rs1867370
13	69741897	A	G	2.82×10^{-3}	1.58×10^{-2}	8.59×10^{-1}	rs2053711
14	75261641	G	A	-5.48×10^{-3}	1.05×10^{-2}	6.02×10^{-1}	rs2111705
14	99706474	G	A	1.33×10^{-2}	1.17×10^{-2}	2.53×10^{-1}	rs10136429
15	74060297	G	A	-8.25×10^{-3}	1.10×10^{-2}	4.53×10^{-1}	rs12440563
15	89989514	C	T	-2.15×10^{-2}	1.34×10^{-2}	1.10×10^{-1}	rs11073864
16	13756549	C	A	8.70×10^{-3}	1.42×10^{-2}	5.41×10^{-1}	rs8053520
18	50597654	G	A	-1.75×10^{-2}	1.14×10^{-2}	1.24×10^{-1}	rs4506980
18	50858019	T	C	-1.13×10^{-2}	1.15×10^{-2}	3.24×10^{-1}	rs1504746
18	53200117	A	G	-9.40×10^{-4}	1.09×10^{-2}	9.31×10^{-1}	rs9636107
18	53456943	A	G	3.43×10^{-3}	1.11×10^{-2}	7.58×10^{-1}	rs4801000
20	44724305	A	G	-1.09×10^{-2}	1.28×10^{-2}	3.95×10^{-1}	rs6131010
20	59002996	C	A	-1.70×10^{-3}	1.14×10^{-2}	8.81×10^{-1}	rs6417358
22	23407063	A	G	9.33×10^{-3}	1.33×10^{-2}	4.82×10^{-1}	rs13054331

	22	46447097	C	T	9.74×10^{-3}	1.21×10^{-2}	4.21×10^{-1}	rs9627391
MDD ↓ COVID-19 infection	1	37169665	C	T	8.25×10^{-3}	4.46×10^{-3}	6.42×10^{-2}	rs218985
	1	37667516	T	C	-2.80×10^{-4}	4.56×10^{-3}	9.52×10^{-1}	rs215814
	1	39551621	C	T	-8.25×10^{-3}	4.72×10^{-3}	8.04×10^{-2}	rs260969
	1	52342427	T	C	-1.38×10^{-3}	4.18×10^{-3}	7.41×10^{-1}	rs1890946
	1	72837490	C	T	-1.82×10^{-5}	4.24×10^{-3}	9.97×10^{-1}	rs11209951
	1	73446704	G	A	-2.04×10^{-3}	4.19×10^{-3}	6.27×10^{-1}	rs12724536
	1	73715233	T	C	-4.17×10^{-3}	4.21×10^{-3}	3.22×10^{-1}	rs11210187
	1	74097006	T	C	6.33×10^{-3}	4.22×10^{-3}	1.33×10^{-1}	rs7524524
	1	173820593	G	A	-8.80×10^{-4}	7.30×10^{-3}	9.04×10^{-1}	rs16846433
	1	177407630	A	G	3.74×10^{-3}	5.58×10^{-3}	5.03×10^{-1}	rs1883905
	2	15450646	T	C	1.06×10^{-3}	4.16×10^{-3}	7.98×10^{-1}	rs10206116
	3	7371919	A	G	-2.56×10^{-3}	4.19×10^{-3}	5.40×10^{-1}	rs17652614
	4	15577738	G	A	5.59×10^{-3}	4.55×10^{-3}	2.20×10^{-1}	rs7665390
	4	90916437	C	T	4.71×10^{-3}	4.30×10^{-3}	2.73×10^{-1}	rs10516854
	4	176869252	G	A	7.50×10^{-3}	5.10×10^{-3}	1.41×10^{-1}	rs41533650
	5	61487083	C	T	5.47×10^{-3}	4.16×10^{-3}	1.89×10^{-1}	rs10057876
	5	87801273	G	T	1.06×10^{-2}	6.55×10^{-3}	1.04×10^{-1}	rs2194026
	5	103729262	C	T	4.96×10^{-3}	4.42×10^{-3}	2.62×10^{-1}	rs4510551
	5	103995368	A	G	1.58×10^{-3}	4.27×10^{-3}	7.11×10^{-1}	rs325485
	5	164484948	T	G	3.67×10^{-3}	4.16×10^{-3}	3.78×10^{-1}	rs4543289
	6	13625413	A	G	-5.62×10^{-3}	5.44×10^{-3}	3.01×10^{-1}	rs10948800
	6	26537801	G	A	6.31×10^{-3}	6.64×10^{-3}	3.42×10^{-1}	rs6920256
	6	27376910	G	A	-4.50×10^{-4}	4.29×10^{-3}	9.16×10^{-1}	rs6938943
	6	27782031	T	G	5.81×10^{-3}	6.88×10^{-3}	3.98×10^{-1}	rs370155
	6	28299687	C	T	1.60×10^{-2}	6.11×10^{-3}	8.79×10^{-3}	rs853676
	6	31094703	C	T	2.68×10^{-3}	7.46×10^{-3}	7.20×10^{-1}	rs3130557
	6	33807091	T	C	-6.18×10^{-3}	4.30×10^{-3}	1.50×10^{-1}	rs6901216
	6	97734271	G	A	-1.62×10^{-3}	4.59×10^{-3}	7.23×10^{-1}	rs9387218
	6	100674018	A	G	-4.18×10^{-3}	4.32×10^{-3}	3.32×10^{-1}	rs6928245
	6	105382415	G	A	-1.83×10^{-3}	4.16×10^{-3}	6.61×10^{-1}	rs12200251

7	2103668	A	G	1.74×10^{-3}	4.74×10^{-3}	7.13×10^{-1}	rs6461115
7	4204008	A	G	7.31×10^{-3}	5.11×10^{-3}	1.52×10^{-1}	rs657323
7	12253088	A	G	-2.22×10^{-3}	4.13×10^{-3}	5.91×10^{-1}	rs6460900
7	82735751	G	A	-2.77×10^{-3}	4.18×10^{-3}	5.08×10^{-1}	rs10264030
7	125867303	A	C	7.79×10^{-3}	5.44×10^{-3}	1.53×10^{-1}	rs1419503
7	126331987	A	G	-1.88×10^{-3}	4.96×10^{-3}	7.04×10^{-1}	rs3808154
9	11145717	T	C	-5.00×10^{-4}	5.46×10^{-3}	9.26×10^{-1}	rs10809359
9	11431990	G	A	-1.12×10^{-2}	5.22×10^{-3}	3.24×10^{-2}	rs10959797
9	11781428	C	A	-8.13×10^{-3}	5.33×10^{-3}	1.27×10^{-1}	rs12377084
9	37223862	A	G	-1.22×10^{-2}	5.30×10^{-3}	2.18×10^{-2}	rs2029646
10	10842639	G	A	-2.86×10^{-3}	4.38×10^{-3}	5.14×10^{-1}	rs2181444
11	31010962	C	T	1.88×10^{-3}	4.20×10^{-3}	6.55×10^{-1}	rs290108
11	88475938	A	G	1.24×10^{-3}	4.40×10^{-3}	7.79×10^{-1}	rs1532547
11	88745439	T	C	6.82×10^{-3}	4.18×10^{-3}	1.03×10^{-1}	rs10830200
11	89007922	C	T	-3.00×10^{-3}	4.49×10^{-3}	5.05×10^{-1}	rs7101897
11	113336172	G	A	-3.40×10^{-3}	4.36×10^{-3}	4.35×10^{-1}	rs11601054
13	31862334	C	A	5.16×10^{-3}	4.83×10^{-3}	2.85×10^{-1}	rs7998271
13	53646138	A	G	-5.33×10^{-3}	4.23×10^{-3}	2.07×10^{-1}	rs2806949
13	53898419	T	G	2.09×10^{-3}	4.57×10^{-3}	6.47×10^{-1}	rs1867370
13	69741897	A	G	2.71×10^{-3}	6.51×10^{-3}	6.77×10^{-1}	rs2053711
14	75261641	G	A	-3.01×10^{-3}	4.10×10^{-3}	4.64×10^{-1}	rs2111705
14	99706474	G	A	-1.09×10^{-3}	4.30×10^{-3}	8.00×10^{-1}	rs10136429
15	74060297	G	A	-3.00×10^{-4}	4.17×10^{-3}	9.43×10^{-1}	rs12440563
15	89989514	C	T	-3.00×10^{-3}	5.21×10^{-3}	5.65×10^{-1}	rs11073864
16	13756549	C	A	2.66×10^{-3}	5.71×10^{-3}	6.41×10^{-1}	rs8053520
18	50597654	G	A	-1.15×10^{-3}	4.30×10^{-3}	7.90×10^{-1}	rs4506980
18	50858019	T	C	2.78×10^{-3}	4.29×10^{-3}	5.17×10^{-1}	rs1504746
18	53200117	A	G	1.92×10^{-3}	4.11×10^{-3}	6.40×10^{-1}	rs9636107
18	53456943	A	G	1.98×10^{-3}	4.32×10^{-3}	6.46×10^{-1}	rs4801000
20	44724305	A	G	1.86×10^{-3}	4.75×10^{-3}	6.96×10^{-1}	rs6131010
20	59002996	C	A	-1.18×10^{-3}	4.24×10^{-3}	7.80×10^{-1}	rs6417358
22	23407063	A	G	-7.90×10^{-4}	4.78×10^{-3}	8.69×10^{-1}	rs13054331
22	46447097	C	T	-7.60×10^{-4}	4.46×10^{-3}	8.64×10^{-1}	rs9627391

severe COVID-19 ↓ MDD	12	113357193	A	G	1.40×10^{-3}	1.84×10^{-3}	4.48×10^{-1}	rs10774671
	19	50882619	C	T	3.13×10^{-3}	1.84×10^{-3}	8.90×10^{-2}	rs1405655
	1	155172725	C	T	1.84×10^{-3}	1.85×10^{-3}	3.22×10^{-1}	rs35154152
	9	136154867	T	G	-4.00×10^{-4}	1.82×10^{-3}	8.27×10^{-1}	rs495828
hospitalized COVID-19 ↓ MDD	10	81706324	G	A	-8.20×10^{-4}	1.84×10^{-3}	6.56×10^{-1}	rs721917
	11	34528766	T	C	-1.63×10^{-3}	1.83×10^{-3}	3.74×10^{-1}	rs766826
	12	113374748	T	C	1.43×10^{-3}	1.84×10^{-3}	4.38×10^{-1}	rs10774679
	19	50882619	C	T	3.13×10^{-3}	1.84×10^{-3}	8.90×10^{-2}	rs1405655
	1	48197570	C	T	-7.20×10^{-4}	1.85×10^{-3}	6.99×10^{-1}	rs12567716
	20	40702071	A	G	5.61×10^{-3}	1.85×10^{-3}	2.39×10^{-3}	rs3746539
	2	60707894	A	G	3.82×10^{-4}	1.86×10^{-3}	8.37×10^{-1}	rs7579014
	6	145026256	A	G	-1.29×10^{-3}	1.88×10^{-3}	4.92×10^{-1}	rs9497053
	9	136149229	C	T	-1.23×10^{-3}	1.84×10^{-3}	5.04×10^{-1}	rs505922
COVID-19 infection ↓ MDD	12	113357193	A	G	1.40×10^{-3}	1.84×10^{-3}	4.48×10^{-1}	rs10774671
	19	4719443	A	G	1.39×10^{-3}	1.83×10^{-3}	4.45×10^{-1}	rs2109069
	20	40715182	G	T	1.37×10^{-3}	1.83×10^{-3}	4.55×10^{-1}	rs2143004
	2	60729294	A	G	-2.02×10^{-5}	1.84×10^{-3}	9.91×10^{-1}	rs10184550
	3	101503765	A	G	-1.78×10^{-3}	1.83×10^{-3}	3.32×10^{-1}	rs4342086
	4	103188709	T	C	5.33×10^{-3}	1.80×10^{-3}	3.02×10^{-3}	rs13107325
	9	136149229	C	T	-1.23×10^{-3}	1.84×10^{-3}	5.04×10^{-1}	rs505922
	9	136194595	C	T	5.00×10^{-4}	1.83×10^{-3}	7.85×10^{-1}	rs17150482

Supplementary Table S6. Heterogeneity tests for bidirectional Mendelian randomization analysis.

**Direction**	**Q**	**Q_df**	**Q_pval**
MDD → severe COVID-19	66.41	62	0.33
MDD → hospitalized COVID-19	66.96	62	0.31
MDD → COVID-19 infection	62.15	62	0.47
severe COVID-19 → MDD	4.46	3	0.22
hospitalized COVID-19 → MDD	11.59	8	0.17
COVID-19 infection → MDD	9.44	7	0.22

Supplementary Table S7. Tests for directional horizontal pleiotropy.

**Direction**	**egger_intercept**	**SE**	***p*-value**
MDD → severe COVID-19	2.16×10^{-2}	2.72×10^{-2}	4.30×10^{-1}
MDD → hospitalized COVID-19	3.76×10^{-3}	1.57×10^{-2}	8.11×10^{-1}
MDD→COVID-19 infection	-9.81×10^{-3}	5.75×10^{-3}	9.32×10^{-2}
severe COVID-19 → MDD	-7.13×10^{-3}	3.87×10^{-3}	2.07×10^{-1}
hospitalized COVID-19 → MDD	-4.22×10^{-3}	3.46×10^{-3}	2.62×10^{-1}
COVID-19 infection → MDD	-1.51×10^{-3}	1.73×10^{-3}	4.16×10^{-1}
